# Expression pattern of secretory‐cell‐related transcriptional signatures in colon adenocarcinomas defines tumor microenvironment characteristics and correlates with clinical outcomes

**DOI:** 10.1002/1878-0261.13338

**Published:** 2022-11-22

**Authors:** Rui Zhou, Lingbo Li, Shaoyan Xi, Yue Zhang, Zhihong Liu, Dongqiang Zeng, Huiying Sun, Jianhua Wu, Ling Wang, Min Shi, Jianping Bin, Yulin Liao, Wangjun Liao

**Affiliations:** ^1^ Department of Oncology, Nanfang Hospital Southern Medical University Guangzhou China; ^2^ Guangdong Province Key Laboratory of Molecular Tumor Pathology Guangzhou China; ^3^ Department of Pathology, Sun Yat‐sen University Cancer Center, State Key Laboratory of Oncology in South China Collaborative Innovation Center for Cancer Medicine Guangzhou China; ^4^ Department of Cardiology, Nanfang Hospital Southern Medical University Guangzhou China

**Keywords:** chemotherapy, colon cancer, immunotherapy, molecular subtype, prognosis, secretory cells

## Abstract

Despite the connection of secretory cells to distinct mucus‐containing colon cancer histological subtypes and the interaction of secretory cells with immune cells in the pathogenesis of intestinal inflammatory diseases, whether the secretory cell signatures are associated with tumor microenvironment (TME) heterogeneity and can aid in colon cancer patient classification have not been investigated. Here, by performing the principal component analysis and consensus clustering analysis, we identified four distinct expression patterns based on secretory cell signatures which were significantly associated with different clinical behaviors, TME landscape, pathway activation, genomic mutations, and DNA methylation characteristics. Subsequently, a ‘SCS score’ model was constructed. The high SCS score indicated a pattern of ‘secretory cell subtype 2’, which was characterized by stromal infiltration and activation, and predicted poor prognosis and low sensitivity to fluorouracil‐based chemotherapy and immunotherapy, but high sensitivity to PI3K catalytic subunit inhibitors. In conclusion, our study comprehensively uncovered the tumor heterogeneity related to secretory cell signature expression patterns. Moreover, the SCS score can supplement routine histopathological assessments to guide personalized therapeutic strategies in colon cancer patients.

## Graphical abstract

Colon cancer patients could be classified into four subtypes, which were significantly associated with different clinical behaviors, immune landscape, pathway activation, genomic mutations, and DNA methylation characteristics, based on secretory‐cell‐related signatures. Additionally, we established a seven gene scoring model derived from secretory cell subtypes that could be used for prognosis and therapeutic benefit prediction in colon cancer patients.

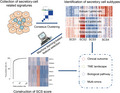

**Abbreviations**
AJDC: adjuvant chemotherapy
CI: confidence interval
CMS: consensus molecular subtypes
COAD: colon adenocarcinoma
CRISPR: Clustered Regularly Interspaced Short Palindromic Repeats
CTRP: Cancer Therapeutics Response Portal
DEGs: deferentially expressed genes
EMT: epithelial–mesenchymal transition
FC: fold change
GEO: Gene Expression Omnibus
HMC: histone modification cluster
ICB: immune checkpoint blockade
ITH: intratumoral heterogeneity
MMR: mismatch repair
MSI: microsatellite instability
MT: mutant type
NEO: neoantigen
NR: nonresponse
OS: overall survival
R: response
Ref: reference
RFS: relapse‐free survival
ROC: receiver operating characteristic
SCS: secretory cell subtype
SIIS: stromal infiltration intensity score
SMG: significant mutated gene
SVMG: significantly varied methylation gene
SYSUCC: Sun Yat‐sen University Cancer Center
TCGA: The Cancer Genome Atlas
TIDE: tumor immune dysfunction and exclusion
TMB: tumor mutation burden
TME: tumor microenvironment
UCSC: University of California Santa
WT: wild type

## 1 Introduction

The disruption of the equilibrium between cellular proliferation, differentiation, and apoptosis in intestinal mucosa, which causes deregulation of the mucosal homeostatic environment is one of the early signs of colon cancer [1, 2]. Among the various cell types in the colonic mucosa, secretory cells represent one of the major populations of differentiated cells, and their role in driving a proportion of colon cancer has received widespread attention due to the close connection of these cells to distinct mucus‐containing colon cancer histological subtypes [3, 4]. The enteroendocrine cells and goblet cells are two main types of secretory cells. Both are derived from LGR5^+^ pluripotent stem cells and their differentiation pathway is controlled by Notch signaling [5, 6]. Physiologically, in response to luminal stimuli, enteroendocrine cells secrete a variety of peptide hormones that act on distant organs and neighboring cells in a traditional endocrine and paracrine manner, respectively [6], whereas goblet cells expel their mucin granules, which combine with water, salt, and other proteins [5]. However, the specific role of these two types of cells present in colon adenocarcinomas in promoting tumorigenesis and development remains unclear and the reported research conclusions are contradictory. For example, although some studies reported that the significant reduction of goblet cell number is a striking feature of colon cancer [7], goblet cells are also considered as key sources of oncogenetic mucin secretion responsible for constitutive activation of growth and survival pathways and downregulation of stress‐induced death pathways [8] and are especially enriched in BRAF mutant colon cancer, a highly aggressive disease subtype [9]. Moreover, a subset of enteroendocrine cells within the tumor expressing the cancer‐associated transcription factor, Brachyury, might represent a population of cells harboring cancer stem cell properties, such as tumor aggressiveness and drug resistance [10]. In addition, mechanistic and biochemical cross‐talk between secretory cells and immune cells during intestinal inflammation, which has been confirmed by a large number of studies [5, 6], also prompts the potential role of secretory cells in shaping tumor microenvironment (TME) heterogeneity in colon cancer. Thus, a more comprehensive profile of the interactions between secretory and immune cells would be helpful for deepening our understanding of cancer immunity.

In view of the clear conservation of core transcriptional profiles in secretory populations between colon cancer and the normal colon [9], in this study: (a) we explored the expression pattern of the transcriptional signatures of enteroendocrine cells and goblet cells based on special feature gene panels for marking secretory cell types curated from a recent study that analyzed the adult large intestine at a single‐cell resolution [11]; (b) we developed novel secretory‐cell‐related transcriptional signature‐based molecular subtypes designated as secretory cell subtype 1 (SCS1), secretory cell subtype 2 (SCS2), secretory cell subtype 3 (SCS3), and secretory cell subtype 4 (SCS4); (c) we established a simple transcriptome score composed of seven genes designated as ‘SCS score’ to quantify these subtypes individually in colon cancer; and (d) we comprehensively assessed the relevance of both our established SCS subtype and SCS score model to TME features, clinicopathological characteristics, and therapeutic responses in multiple independent colon cancer cohorts.

## 2 Materials and methods

### 2.1 Data retrieval and preprocessing

The public transcriptome data of nonmetastatic colon cancer samples (stages I–III) used in the current study were retrospectively collected from the Gene Expression Omnibus (GEO, https://www.ncbi.nlm.nih.gov/geo/) and The Cancer Genome Atlas (TCGA, https://cancergenome.nih.gov/) datasets (Tables S1 and S2). For the GEO dataset, five cohorts with detailed survival data and the same hybridization platform, namely GSE17538, GSE33113, GSE37892, GSE38832, and GSE39582, were downloaded and combined into a meta‐GEO cohort using the ‘sva’ R package [12]. For the TCGA dataset, we downloaded level three ‘HTSeq‐Counts’ data of eligible samples from the ‘TCGA‐COAD’ item of University of California Santa Cruz (UCSC) Xena database (https://xenabrowser.net), and the RNA sequencing data was transformed using the ‘voom’ algorithm after gene symbol transformation to convert count data to values similar to those resulting from microarrays [13]. In addition, RNA sequencing data of 30 fresh samples that were histologically diagnosed with nonmetastatic colon cancer at the Sun Yat‐sen University Cancer Center (SYSUCC, Guangzhou, China, Tables S1 and S2) were collected as an external validation cohort, as we previously described [14, 15]. The experiments were undertaken with the understanding and written consent of each subject. The study methodologies were approved by our institutional review board (NFEC‐2019‐263) and were conducted according to the principles of the Declaration of Helsinki.

### 2.2 Principal component analysis (PCA) algorithm‐based evaluation of secretory cell marker enrichment, TME cell marker enrichment, and biological pathway activation

The enrichment levels of secretory cells, TME cells, and biological pathways were evaluated using the PCA algorithm incorporated in the IOBR package [16] based on predefined signatures and were represented by the calculated PCA scores. The annotated gene sets for secretory cells were collected from the research conducted by Gao et al. which defined three secretory cell types, namely enteroendocrine cells, subtype 1 goblet cells, and subtype 2 goblet cells, by analyzing adult normal intestinal mucosa using single‐cell RNA sequencing [11]. In particular, CHGA was specifically added into the marker list for enteroendocrine cells, while TFF3 and MUC2 were specifically added into the marker lists for the two subtypes of goblet cells before we performed the PCA algorithm calculation. For TME deconvolution, 23 special feature gene panels for myeloid cells, lymphocytes, and stromal cells were curated from the published literature [17, 18]. In addition, 50 hallmark biological pathways and 186 KEGG pathways were collected from the Molecular Signature Database to measure the biological processes of each sample.

### 2.3 Unsupervised clustering of the estimated abundance of secretory cells

Based on the PCA score of signatures of enteroendocrine cells, subtype 1 goblet cells, and subtype 2 goblet cells, we performed unsupervised clustering analysis (K‐means) to comprehensively identify distinct secretory cell enrichment patterns. The ConsensuClusterPlus package incorporates a consensus clustering algorithm and was used to evaluate the clustering stability and select the optimal cluster number [19]. The following parameters were set: maxK = 10, reps = 1000, pItem = 0.95, and pFeature = 1.

### 2.4 Generation of the SCS score

The process used to establish the SCS score was similar to that used in previous studies [14, 15]. First, we analyzed differentially expressed genes (DEGs) between SCS2 and non‐SCS2 patients in the GSE39582 cohort using the limma package [20]. The adjusted *P*‐value for multiple testing was calculated using the Benjamini–Hochberg correction. The significance criterion for determining DEGs was set as an absolute ‘Log2FC’ value > 1 and an adjusted *P*‐value < 0.01. Then, we used the Boruta dimension reduction algorithm to screen the feature genes from 608 DEGs upregulated and 20 DEGs downregulated in the SCS2 subtype, with the following settings: doTrace = 2, maxRuns = 100, ntree = 500. Next, the feature genes selected by the Boruta algorithm were dichotomized based on the optimal cutoff points determined by the survminer package with the maximally selected log‐rank statistics. Univariate Cox analysis was performed on these dichotomous genes to identify prognostic genes. After removing the remaining prognostic genes that were not included in the TCGA‐COAD cohort, Cox regression with least absolute shrinkage and selection operator penalty (LASSO‐Cox) analysis was performed to select the most useful prognostic markers. The final scoring model consisted of seven genes and was defined as follows: SCS score = FAM13C + PRKD1 + AKAP13 + A2M + FSTL1 − ASCL2 − FAM84A.

### 2.5 Therapeutic response prediction

The drug sensitivity to fluorouracil single agent for each sample was predicted using pRRophetic [21], an R package implements a built‐in ridge regression model based on the Cancer Therapeutics Response Portal (CTRP) database, and was qualified as the area under the dose–response curve (AUC), with lower AUC values indicating higher sensitivity. For immune checkpoint blockade (ICB) response prediction, we applied tumor immune dysfunction and exclusion (TIDE) [22] and SubMap algorithms [14]. TIDE was implemented using transcriptome profiles using an online tool (http://tide.dfci.harvard.edu). SubMap is an algorithm that reveals common subtypes between independent datasets and is achieved through the online module of the GenePattern website (https://cloud.genepattern.org/). Before SubMap analysis, we analyzed the DEGs between ICB‐responsive and non‐responsive samples in the IMvigor210 dataset, which contains transcriptomic data from patients with metastatic urothelial cancer treated with anti‐PD‐L1 agents (atezolizumab, IMvigor dataset, retrieved via r software using the IMvigor210CoreBiologies package [23]). DEGs with an absolute ‘Log2FC’ value > 0.5 were selected as marker genes for the comparison of similarity between SCS subtypes and ICB response status.

### 2.6 Genetic mutation analysis and DNA methylation analysis

The genetic mutation file was downloaded using the TCGAbiolinks package [24] and the significant cancer mutated genes (SMGs) were identified by the MutSigCV algorithm (*q* < 0.05) [25]. Then, we selected the SMGs whose total mutation frequency ranked in the top 100 and analyzed the distribution of effective mutations of these SMGs among the four SCS subtypes using the chi‐square test. For DNA methylation analysis, we downloaded level three DNA methylation (Methylation450k) data from the UCSC Xena database and preprocessed the data using the ChAMP package as previously described [26]. We assigned DNA methylation values for each gene with the median beta value of the probes mapped to the promoter region and the 1000 genes with the largest variability in methylation value (β values) were selected as candidates to explore the differential methylated genes among the four SCS subtypes using the Kruskal–Wallis test. Meanwhile, we also tested the transcriptional expression variation of the above candidate genes in the four SCS subtypes and the correlation between the transcriptional expression level and methylation level of each gene. The adjusted *P*‐value for multiple testing was calculated using the Benjamini–Hochberg correction. Finally, we defined significantly varied methylation genes (SVMGs) that related to SCS subtype based on the following criteria: both the methylation value and the corresponding transcriptional expression value of SVMGs were significantly varied among the four SCS subtypes (adjusted *P*‐value < 0.05), and there was a significant negative correlation between the methylation value and the corresponding transcriptional expression value of each SVMG (adjusted *P*‐value < 0.05).

### 2.7 Statistical analysis

Statistical analysis was performed using R software version 4.0.2 or SPSS version 25.0 (IBM Corp., Armonk, NY, USA). Two‐tailed Student's *t*‐test and Mann–Whitney *U* test were performed to compare the differences in normally distributed and non‐normally distributed continuous variables between the two groups, respectively. Kruskal–Wallis (non‐parametric) and one‐way ANOVA tests (parametric methods) were used for comparisons of more than two groups. We used chi‐squared and Fisher's exact tests to analyze the difference between categorical variables. The Pearson's correlation test and Spearman's rank correlation test were used to evaluate correlations between continuous variables when appropriate. For survival analysis, the Cox regression hazard model and Kaplan–Meier method with the log‐rank test were used where necessary. The best cutoff values for each continuous prognostic marker were calculated using the Survminer package. All *P*‐values were two‐tailed, and statistical significance was set at *P* < 0.05, unless otherwise noted.

## 3 Results

### 3.1 The TME, biological, and clinical characteristics associated with expression level of secretory cell‐related transcriptional signatures

**Fig. 1:**
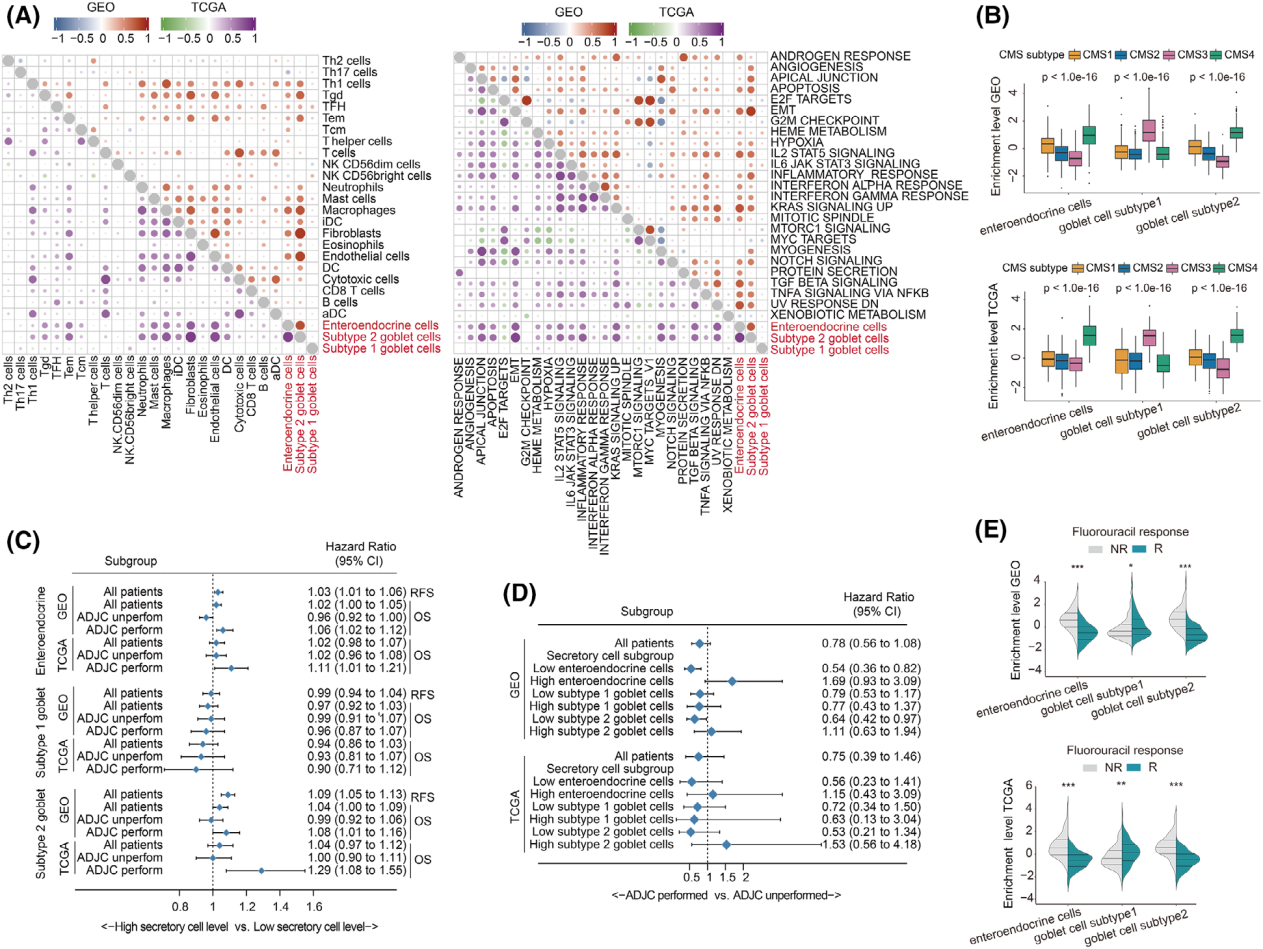
The clinical relevance, tumor microenvironment characteristics, and biological function characteristics of PCA score of secretory cells. (A) Heatmaps of the association between the PCA scores of secretory cells, tumor microenvironment cells (left), and hallmark pathway activation (right) in the meta‐GEO (upper right, 990 patients) and TCGA‐COAD (lower left, 382 patients) cohorts. (B) Boxplot of the PCA scores of secretory cells among patients with different CMS molecular subgroups in the meta‐GEO (upper, 713 patients) and TCGA‐COAD (down, 382 patients) cohorts. Boxes represent 25–75% of values, lines in boxes represent median values, whiskers represent 1.5 interquartile ranges, and black dots represent outliers. One‐way ANOVA was used to determine significance. (C) Forest plots of the associations between PCA scores of secretory cells and patient survival in various subgroups in the meta‐GEO (RFS analysis, 990 patients; OS analysis, 678 patients) and TCGA‐COAD (370 patients) cohorts. Unadjusted hazard ratios (boxes) and 95% confidence intervals (horizontal lines) are depicted. (D) Forest plots of benefits of adjuvant chemotherapy in various subgroups in the GSE39582 (502 patients) and TCGA‐COAD cohorts (323 patients). Unadjusted hazard ratios (boxes) and 95% confidence intervals (horizontal lines) are depicted. (E) Violin plot of distribution of PCA scores of secretory cells among patients with different fluorouracil responses in the GSE39582 (335 patients) and TCGA‐COAD (249 patients) cohorts. Student's *t*‐test was used to determine significance. *P < 0.05, **P < 0.01, ***P < 0.001; aDC, activated dendritic cell; ADJC, adjuvant chemotherapy; CI, confidence interval; CMS, consensus molecular subtypes; DC, dendritic cell; iDC, immature dendritic cell; NK, natural killer; NR, nonresponse; OS, overall survival; R, response; RFS, relapse‐free survival; Tcm, T central memory; Tem, T effector memory; TFH, T follicular helper; Tgd, T gamma delta; Th, T helper.

Figure S1 represents the workflow of this study. We first explored the role of the secretory‐cell‐related transcriptional signature expression level in the tumors of colon cancer patients. As shown in Fig. 1A, in both the meta‐GEO and TCGA‐COAD cohorts, there were significant positive correlations between the expression level of enteroendocrine cell and subtype 2 goblet cell signatures, while the expression level of the subtype 1 goblet cell signature was independent of the above two cell type signatures. In terms of the correlations between PCA scores of secretory cell‐related signatures and the TME cell‐related signatures (Fig. 1A, left), we found that the PCA scores of both enteroendocrine cell and subtype 2 goblet cell signatures showed significant positive correlations with the scores of stromal cell and some myeloid cell types, such as fibroblasts, endothelial cells, macrophages, and mast cells, in both the meta‐GEO and TCGA‐COAD cohorts. Correspondingly, there were also highly significant positive correlations between the PCA score of enteroendocrine cell and subtype 2 goblet cell signatures and the activation level of stromal‐related pathways, including epithelial–mesenchymal transition (EMT) and TGFβ signaling (Fig. 1A, right). Interestingly, the gene sets of IL2‐STAT5 signaling, inflammatory response pathway, and KRAS signaling were also significantly elevated in tissues with highly enriched gene markers of enteroendocrine cells and subtype 2 goblet cells. The following KEGG analyses not only confirmed the positive correlation between the activation level of stromal‐related pathways and the PCA scores of enteroendocrine cells and subtype 2 goblet cells, but also revealed significant positive relationships between estimated scores of the subtype 1 goblet cell signature and multiple cell metabolism pathways (Table S3). The comparison of enrichment degree of secretory cell‐related signatures among different consensus molecular subtypes (CMS) [27] in both the meta‐GEO and TCGA‐COAD cohorts revealed that PCA scores of enteroendocrine cell and subtype 2 goblet cell signatures were the highest in the tissues of CMS4 patients, which mostly represent stromal/mesenchymal phenotypes; the score of subtype 1 goblet cell signature was highest in the tissues of CMS3 patients, a subtype characterized by prominent metabolic activation (Fig. 1B).

Next, we explored the clinical significance of the expression level of secretory cell‐related signatures in colon cancer patients. As shown in Fig. 1C, unlike subtype 1 goblet cells, whose PCA score has no significant correlation with patient survival, higher levels of both enteroendocrine cell and subtype 2 goblet cell signatures indicate a significantly higher risk of recurrence. In overall survival (OS), we found that negative correlations of the PCA scores of enteroendocrine cell and subtype 2 goblet cell signatures with the OS time were only significant in patients who underwent adjuvant chemotherapy (ADJC), suggesting that the presence of enteroendocrine cells and subtype 2 goblet cells may be associated with chemotherapy resistance (Fig. 1C). To validate this hypothesis, we analyzed the relationship between the PCA score of secretory cell‐related signatures and chemotherapy benefit in both the GSE39582 and TCGA‐COAD cohorts (Fig. 1D). The results showed that among the patients with highly enriched enteroendocrine cell and subtype 2 goblet cell signatures (the PCA score ranked in the top 1/3, number of patients in high enrichment group in GSE39582: 167 and in TCGA‐COAD: 127), ADJC increased mortality risk (Fig. 1D). Meanwhile, the violin diagram also revealed that the enrichment levels of enteroendocrine cell and subtype 2 goblet cell signatures were significantly higher in the fluorouracil‐nonresponsive group than in the fluorouracil‐responsive group, while the subtype 1 goblet cell signature was significantly enriched in the fluorouracil‐responsive group (Fig. 1E). In summary, these results indicate that enrichment level of secretory cells, especially enteroendocrine cells and subtype 2 goblet cells, may be potential predictors of prognosis and chemotherapeutic response in colon cancer.

### 3.2 Identification of secretory‐cell‐related signature expression pattern

**Fig. 2:**
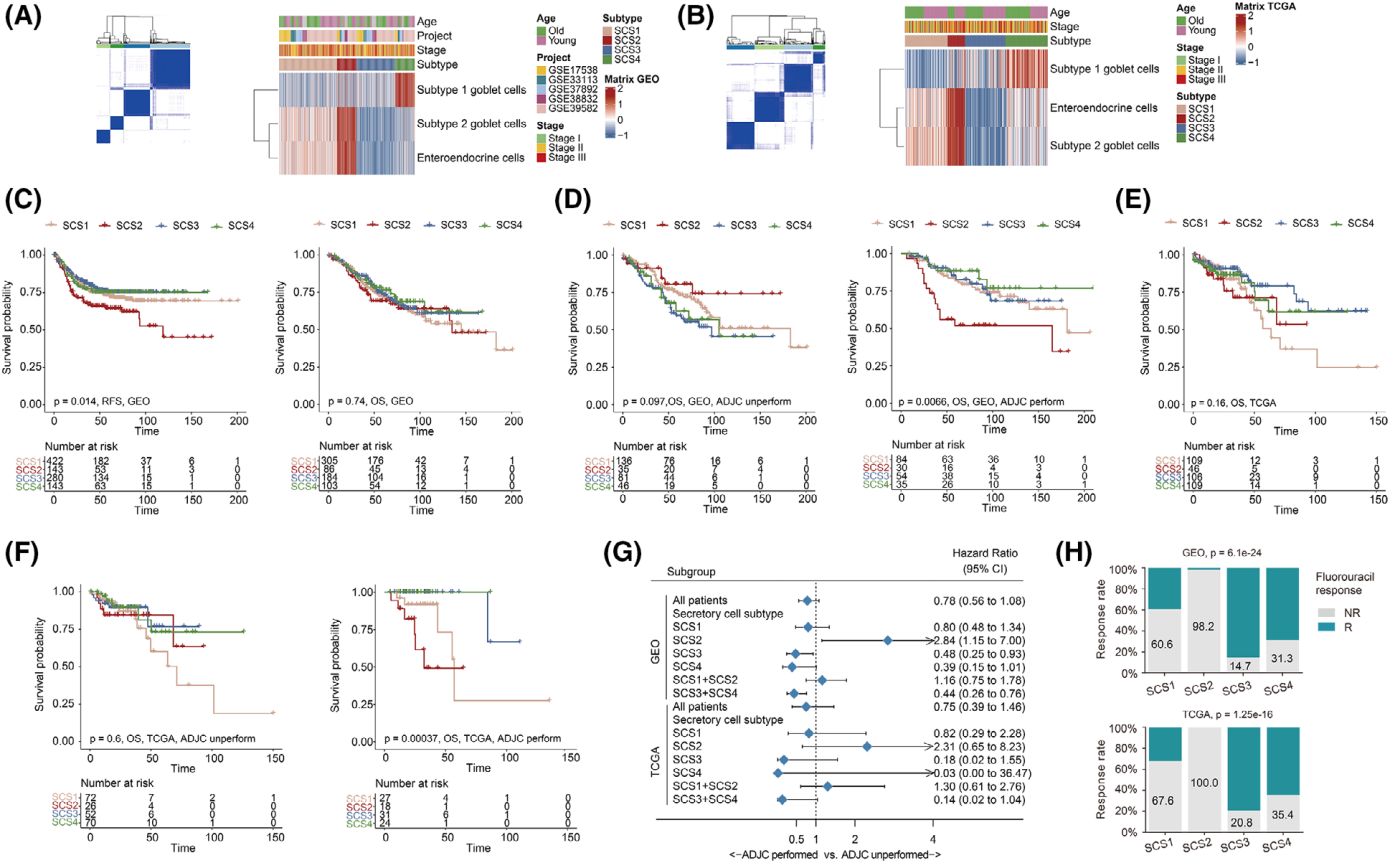
Consensus clustering based on PCA scores of secretory cell‐related signatures in colon cancer. (A, B) Consensus matrices (left) and Heatmaps (right) demonstrating the expression patterns of secretory cell‐related signatures in patients with colon cancer identified by the unsupervised clustering analysis of PCA scores of three types of secretory cells in the meta‐GEO (A, 990 patients) and TCGA‐COAD (B, 382 patients) cohort. Cohort details and SCS subtypes are used as annotations. (C) Kaplan–Meier curves of relapse‐free survival (left) and overall survival (right) according to SCS subtypes in the meta‐GEO cohort (RFS analysis, 990 patients; OS analysis, 678 patients). (D) Kaplan–Meier curves of overall survival according to SCS subtypes in the patient subgroup without (left) or with adjuvant chemotherapy (right) in the GSE39582 cohort (502 patients). (E) Kaplan–Meier curves of overall survival according to SCS subtypes in the TCGA‐COAD cohort (370 patients). (F) Kaplan–Meier curves of overall survival according to SCS subtypes in the patient subgroup without (left) or with adjuvant chemotherapy (right) in the TCGA‐COAD cohort (323 patients). (G) Forest plots of benefits of adjuvant chemotherapy in different SCS subtypes in the GSE39582 (502 patients) and TCGA‐COAD (323 patients) cohorts. Unadjusted hazard ratios (boxes) and 95% confidence intervals (horizontal lines) are depicted. (H) Bar charts summarize the proportions of patients in fluorouracil‐response group and those in nonresponse group within and across different SCS subtypes (GSE39582: 335 patients; TCGA‐COAD: 249 patients). ADJC, adjuvant chemotherapy; CI, confidence interval; NR, nonresponse; OS, overall survival; R, response; RFS, relapse‐free survival; SCS, secretory cell subtype.

To identify the secretory‐cell‐related signature expression patterns in colon cancer, we employed an unsupervised k‐means clustering algorithm based on the PCA scores of enteroendocrine cell, subtype 1 goblet cell, and subtype 2 goblet cell signatures in the meta‐GEO and TCGA‐COAD cohorts in the meta‐GEO and TCGA‐COAD cohorts. As shown in Fig. 2A,B, four distinct expression patterns of secretory cell‐related signatures achieved the best clustering efficacies, and patients were classified into SCS1, SCS2, SCS3, and SCS4 subtypes, accordingly. Among them, samples of SCS1, SCS2, and SCS3 were characterized by moderate, highest, and lowest enrichment levels, respectively, of enteroendocrine cell and subtype 2 goblet cell signatures, while SCS4 showed intensive enrichment of the subtype 1 goblet cell signature. Subsequent survival analyses showed that patients with the SCS2 subtype had a significantly shorter relapse‐free survival (RFS) than those with other SCS subtypes (*P* = 0.014, Fig. 2C). As for OS, similar to the results of univariate survival analysis of PCA scores of enteroendocrine cell and subtype 2 goblet cell signatures, SCS2 only exhibited a significantly worse prognosis than other subtypes in patients subgroups who received ADJC in both the GSE39582 (*P* = 0.007, Fig. 2C,D) and TCGA‐COAD cohorts (*P* < 0.001, Fig. 2E,F). In terms of chemotherapy benefit, the forest diagram (Fig. 2G) and stacked histogram (Fig. 2H) showed that ADJC performance increased mortality risk more than twofold in SCS2 patients in both the GSE39582 [hazard ratio (HR) = 2.84, 95% confidence interval (CI) = 1.15–7.00] and TCGA‐COAD (HR = 2.31, 95%CI = 0.65–8.23) cohorts, and the fluorouracil‐response rate was also the lowest in patients with the SCS2 subtype. In contrast, SCS3 patients with the lowest enrichment level of enteroendocrine cell and subtype 2 goblet cell signatures showed the highest response rate to fluorouracil. Taken together, these data indicate that the clinical characteristics of SCS2 patients are mainly attributed to the high enrichment of enteroendocrine cell and subtype 2 goblet cell signatures in tumor tissue.

### 3.3 TME and biological characteristics of different secretory cell patterns

**Fig. 3:**
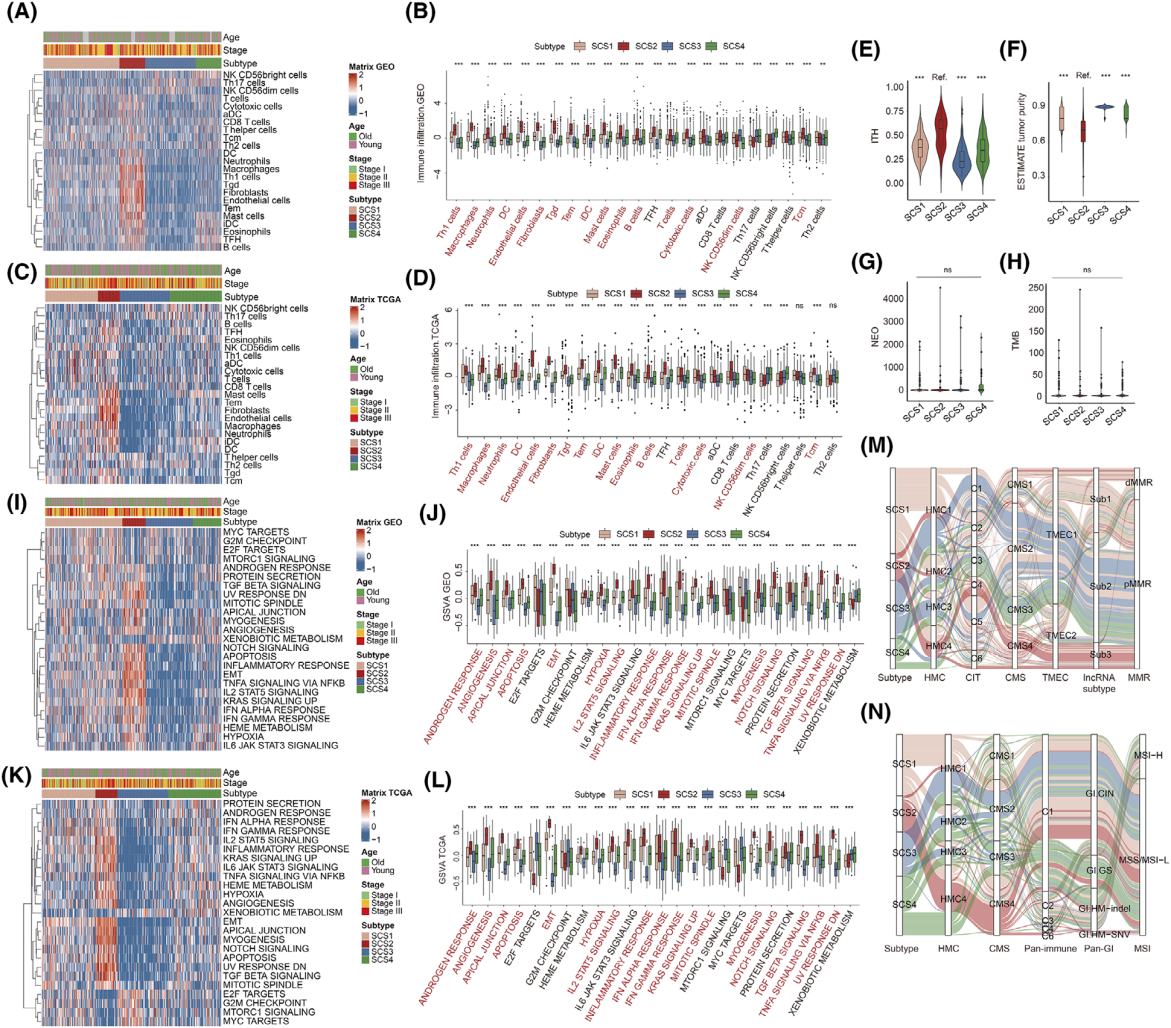
Tumor microenvironment and biological function characteristics of distinct SCS subtypes. (A–D) Heatmaps (A, C) and box plots (B, D) show the results of tumor microenvironment landscape in the four SCS subtypes in the meta‐GEO (A, B, 990 patients) and TCGA‐COAD (C, D, 382 patients) cohorts. Cohort details and SCS subtypes are used as sample annotations of the heatmaps. Boxes represent 25–75% of values, lines in boxes represent median values, whiskers represent 1.5 interquartile ranges, and black dots represent outliers. Red terms in the box plots indicate that the corresponding items have the highest level in SCS2 patients. One‐way ANOVA was used to determine significance. (E–H) Violin plot of intratumoral heterogeneity levels (E, 237 patients), tumor purity (F, 304 patients), neoantigen (G, 307 patients), and tumor mutation burden (H, 304 patients) in the four SCS subtypes in the TCGA‐COAD cohort. Boxes inside the violins represent 25–75% of values, lines in boxes represent median values, whiskers represent 1.5 interquartile ranges, and black dots represent outliers. One‐way ANOVA was used to determine significance. (I–L) Heatmaps (I, K) and box plots (J, L) show the biological pathway activation status based on ‘hallmark gene sets’ in the four SCS subtypes in the meta‐GEO (I, J, 990 patients) and TCGA‐COAD (K, L, 382 patients) cohort. Cohort details and SCS subtypes are used as sample annotations of the heatmaps. Boxes represent 25–75% of values, lines in boxes represent median values, whiskers represent 1.5 interquartile ranges, and black dots represent outliers. Red terms in the box plot indicate that the corresponding items have the highest level in SCS2 patients. One‐way ANOVA was used to determine significance. (M, N) Sankey diagram of SCS subtypes in groups with different molecular subtypes in the GSE39582 (upper, 412 patients) and TCGA‐COAD (down, 201 patients) cohorts. *P < 0.05, **P < 0.01, ***P < 0.001; CMS, consensus molecular subtypes; HMC, histone modification cluster; ITH, intratumoral heterogeneity; NEO, neoantigen; ns, not significant; Ref, reference; SCS, secretory cell subtype; SNV, single‐nucleotide variant; TMB, tumor mutation burden; TMEC, tumor microenvironment cluster.

Since the PCA scores of secretory cell‐related signatures have been shown to be correlated with the expression levels of various types of TME cell‐related signatures, we further compared TME cell‐related signatures among the SCS subtypes. Based on the PCA algorithm, we found that the SCS subtypes displayed different enrichments for TME cell‐related signatures (Fig. 3A–D). In SCS2, PCA scores of multiple stromal cells and innate immune cells with immunosuppressive properties, such as fibroblasts, endothelial cells, macrophages, neutrophils and mast cells, showed the highest level, suggesting that the TME of SCS2 samples might be classified as the feature of the ‘immune‐excluded’ phenotype (a phenotype characterized by stromal cell infiltration so that the cytotoxic cells are retained in the stroma surrounding tumor cell nests rather than in the parenchyma [28]). In accordance with this, the value of our previously developed SIIS model [14], a 31 gene signature for effectively quantifying stromal cell components of the TME in colon cancer patients, was also the highest for the SCS2 subtype in both the meta‐GEO and TCGA‐COAD cohorts (Fig. S2). In contrast, the TME feature of SCS3 was similar to the ‘immune‐desert’ phenotype because most TME cell signatures had the lowest enrichment level in the SCS3 samples. PCA scores of almost all TME cell types showed moderate levels in the SCS1 samples. In addition, we found that compared with other subtypes, SCS2 had the highest intratumoral heterogeneity (Fig. 3E) and the lowest tumor purity (Fig. 3F). However, there was no significant difference in the levels of tumor mutation burden (Fig. 3G) and the number of neoantigens (Fig. 3H) among the different SCS subtypes.

We then investigated the pathway activation related to each SCS subtype by performing a PCA algorithm against the hallmark and KEGG gene sets. As shown in Fig. 3I–L, stromal‐related pathways and inflammation‐related pathways were highly activated in SCS2 patients but significantly inhibited in SCS3 patients. Moreover, KRAS signaling was highly activated in SCS2 patients, indicating that KRAS signaling might play an important role in the progression of SCS2 tumors. Finally (Fig. 3M,N), we found that SCS2 patients were mainly concentrated in multiple subtypes displaying stromal properties, including HMC4 [15], C4 (CIT) [29], CMS4 [27], TMEC2 [30], Sub 3 [31], and C6 (Pan‐Immune, TCGA) [32] subtypes, while almost all SCS4 patients were enriched in CMS3. However, the distributions of mismatch repair (MMR) or microsatellite instability (MSI) status of patients with different SCS subtypes in GSE39582 and TCGA‐COAD cohorts were inconsistent. In conclusion, these results suggest that SCS2 patients mainly exhibit stromal‐related TME and biological features, which may be one of the reasons for chemoresistance in this subtype of patients.

### 3.4 Multi‐omics analysis of different SCS subtypes

**Fig. 4:**
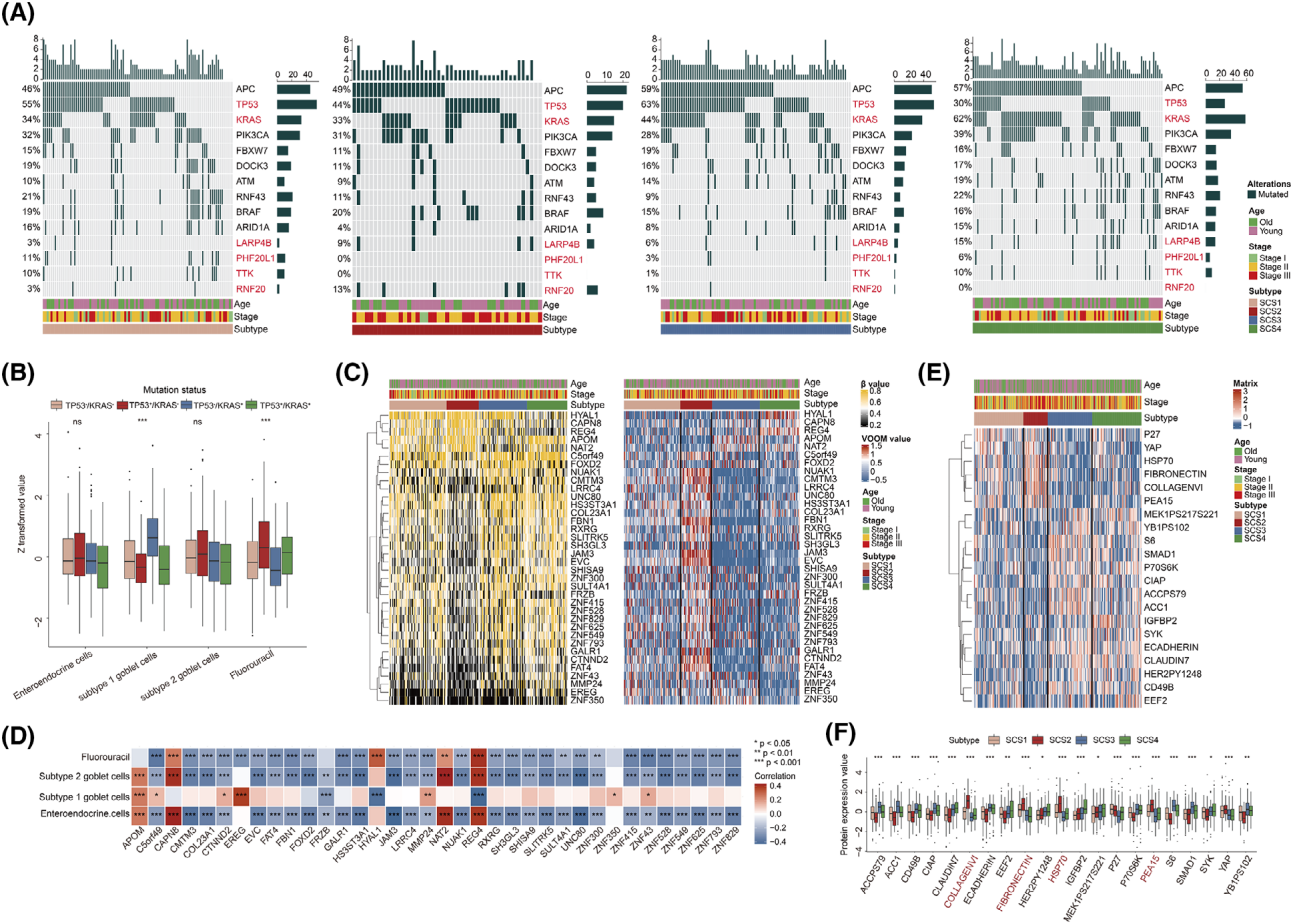
SCS subtype‐related genomic alterations and methylation characteristics. (A) Oncoprints depicted the somatic mutations of significant mutated genes in the context of four SCS subtypes in the TCGA‐COAD cohort (350 patients). Cohort details and SCS subtypes are used as sample annotations of the heatmaps. (B) Box plots show the distribution of PCA scores of secretory cells and predicted AUC value of fluorouracil in different mutation status of gene combination in the TCGA‐COAD cohort (350 patients). Boxes represent 25–75% of values, lines in boxes represent median values, whiskers represent 1.5 interquartile ranges, and black dots represent outliers. One‐way ANOVA was used to determine significance. (C) Heatmaps of significantly varied methylation genes among four SCS subtypes at methylation level (left) and transcriptome level (right) in the TCGA‐COAD cohort (243 patients). (D) Correlation matrix showing the correlations of PCA scores of secretory cells and predicted AUC value of fluorouracil with the methylation values of significantly varied methylation genes in the TCGA‐COAD cohort (243 patients). Spearman's correlation test was used to determine significance. (E) Heatmap exhibited the landscape of differentially expressed proteins in four SCS subtypes. (F) Boxplot of protein expression in four SCS subtypes in the TCGA‐COAD cohort (282 patients). Boxes represent 25–75% of values, lines in boxes represent median values, whiskers represent 1.5 interquartile ranges, and black dots represent outliers. One‐way ANOVA was used to determine significance. *P < 0.05, **P < 0.01, ***P < 0.001; SCS, secretory cell subtype.

To better understand the biological characteristics of different SCS subtypes, we conducted multi‐omics analysis in the TCGA‐COAD cohort. First, regarding distribution of effective mutation of SMGs among different SCS subtypes, we found that KRAS had the highest mutation rate in the SCS4 subtype in the entire, MSI‐high (MSI‐H), and microsatellite stability/MSI‐low (MSS/MSI‐L) patient cohorts (Fig. 4A and Fig. S3A,B) and KRAS mutation was highly correlated with the PCA score of the subtype 1 goblet cells signature (Fig. 4B and Fig. S3C,D). Interestingly, the mutation rate of TP53 in SCS4 subtype was significantly lower than that in other SCS subtypes in the entire and MSS/MSI‐L cohort (Fig. 4A and Fig. S3B) and TP53 mutation was highly correlated with the predicted fluorouracil AUC value (Fig. 4B and Fig. S3C,D). It is worth noting that in the MSS/MSI‐L cohort, BRAF mutation was significantly enriched in SCS2 patients compared with other SCS subtypes (Fig. S3B).

In addition to genomic mutations, we also explored the differences in gene promoter methylation levels among SCS subtypes. As shown in Fig. 4C and Table S4, a total of 36 SVMGs were identified. Among these genes, HYAL1, CAPN8, REG4 and NAT2 are highly methylated with low transcriptional expression, while other genes are low methylated with high transcriptional expression in the SCS2 subtype, suggesting that methylation of these genes may play an important role in the biological characteristics of SCS2 patients. The associations of the methylation level of these SVMGs to PCA scores of secretory cell‐related signatures and predicted fluorouracil AUC values are shown in Fig. 4D.

Finally, we downloaded proteomic data from TCGA‐COAD cohorts. Consistent with the results of transcriptome analysis (Fig. 4E,F), the protein expression of mesenchymal markers such as fibronectin and type IV collagen were highly expressed, while that of E‐cadherin, the epithelial marker, was significantly decreased in the SCS2 subtype, which further confirmed that there was a high activation of stromal pathways in SCS2 at the protein level.

### 3.5 Construction of the SCS score and exploration of its clinical relevance in the meta‐GEO cohort

**Fig. 5:**
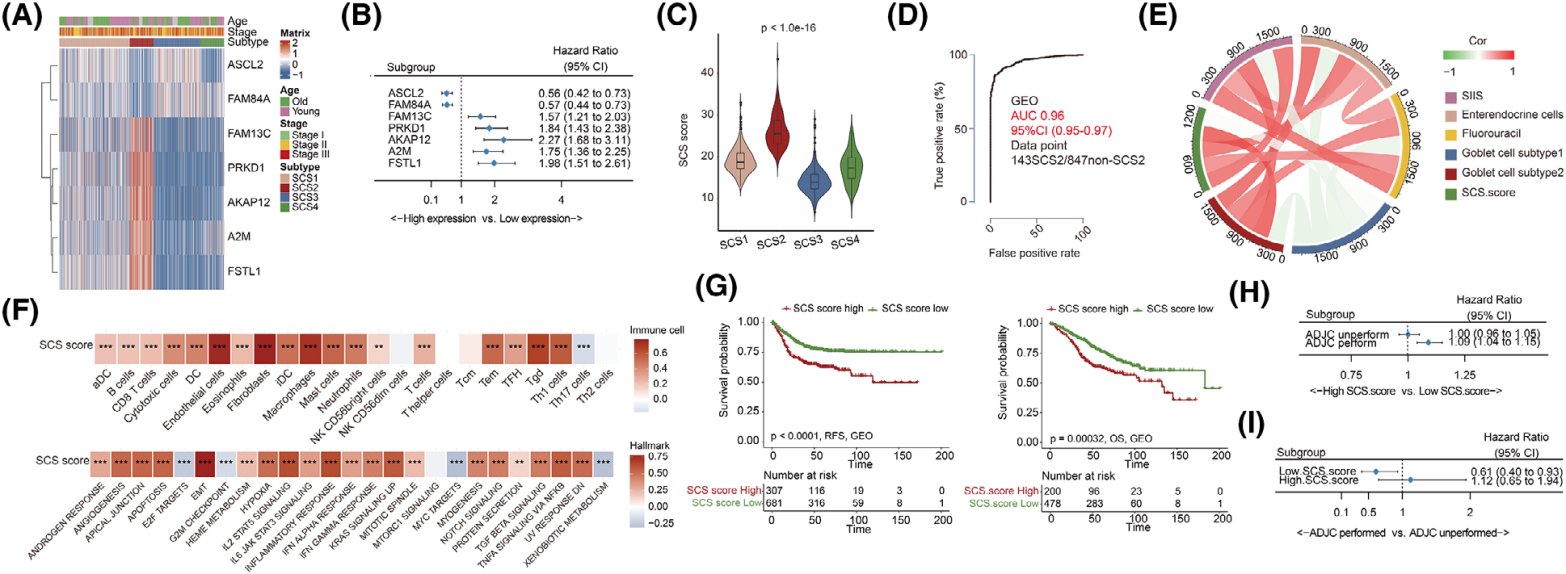
Construction and exploration of the SCS score in the meta‐GEO cohort. (A) Heatmap shows the expression of genes consisting of SCS score in four SCS subtypes of the meta‐GEO cohort (990 patients). Cohort details and SCS subtypes are used as sample annotations of the heatmaps. (B) Forest plots of associations between the expression of genes consisting of SCS score and patient relapse‐free survival in the meta‐GEO cohort (990 patients). Unadjusted hazard ratios (boxes) and 95% confidence intervals (horizontal lines) are depicted. (C) Violin plot of SCS score value in four SCS subtypes in the meta‐GEO cohort (990 patients). Boxes inside the violins represent 25–75% of values, lines in boxes represent median values, whiskers represent 1.5 interquartile ranges, and black dots represent outliers. One‐way ANOVA was used to determine significance. (D) Receiver operating characteristics curve of the SCS score model in the meta‐GEO cohort (990 patients). (E) The correlation chord chart showing the mutual correlation between SIIS value, PCA scores of secretory cells, SCS score, and predicted AUC value of fluorouracil in the GSE39582 cohort (502 patients). (F) Correlation matrix showing the correlations of PCA scores of tumor microenvironment cells (upper) and hallmark pathways (bottom) with the SCS score in the meta‐GEO cohort (990 patients). Pearson's correlation test was used to determine significance. (G) Kaplan–Meier curves of relapse‐free survival (left, 990 patients) and overall survival (right, 678 patients) in the meta‐GEO cohort according to the SCS score; (H) Forest plots of associations between the SCS score and overall survival in subgroups stratified by adjuvant chemotherapy conduction in the GSE39582 cohort (502 patients). Unadjusted hazard ratios (boxes) and 95% confidence intervals (horizontal lines) are depicted. (I) Forest plots of benefits of adjuvant chemotherapy in different SCS score groups in the GSE39582 cohort. Unadjusted hazard ratios (boxes) and 95% confidence intervals (horizontal lines) are depicted. *P < 0.05, **P < 0.01, ***P < 0.001; ADJC, adjuvant chemotherapy; AUC, area under ROC curve; CI, confidence interval; EMT, epithelial–mesenchymal transition; OS, overall survival; RFS, relapse‐free survival; SCS, secretory cell subtype; SIIS, stromal cell infiltration intensity.

Because patients within the SCS2 subtype have poor prognosis and could not benefit from fluorouracil treatment, we believe that it was necessary to develop a simple scoring tool to accurately identify such patients in clinical practice and to assist with TNM staging and guide ADJC performance in colon cancer. Through a series of steps depicted in Fig. S1, we identified 628 DEGs (Table S5) in the GSE39582 cohort, and successively used Boruta dimension reduction and LASSO‐Cox regression analysis to establish a scoring model composed of seven genes, termed as the SCS score (Fig. 5A). The summaries for these seven genes are listed in Table S6. Among these genes, ASCL2 and FAM84A were downregulated in the SCS2 subtype and their expression levels were significantly negatively correlated with the risk of relapse. The other five genes were highly expressed in SCS2 patients, and their overexpression was significantly associated with worse RFS (Fig. 5B). The violin plot (Fig. 5C) showed that the median SCS score was the highest in SCS2, followed by the SCS1 subtype, and lowest in SCS3 patients. The receiver operating characteristic (ROC) curve analysis further demonstrated that the SCS score was a reliable index to distinguish patients of SCS2 subtype with a diagnostic accuracy of 0.96 (Fig. 5D). Through correlation tests (Fig. 5E), we found that the SCS score was highly positively correlated with the PCA scores of enteroendocrine cells and subtype 2 goblet cells, AUC of fluorouracil response, and SIIS value, indicating that the SCS score is an effective index that can reflect the enrichment degree of both enteroendocrine cell and subtype 2 goblet cell signatures and is associated with fluorouracil resistance. Moreover, the SCS score was markedly positively correlated with stromal pathway and stromal cell signature expression (Fig. 5F) and was also significantly elevated in dMMR patients (Fig. S4A), which was consistent with the results for the SCS2 subtype.

Next, we explored the role of the SCS score in indicating prognosis and chemotherapy benefit in patients with colon cancer. We first used the survminer package to divide patients into high‐ and low‐SCS score groups and found that the group with low SCS scores had a significantly higher RFS (HR = 1.92, 95% CI = 1.49–2.48, Fig. 5G, left) and OS (HR = 1.66, 95%CI = 1.25–2.21, Fig. 5G, right). Further analysis of SCS score as a continuous variable showed that the SCS score was an independent prognostic factor for RFS (HR = 1.05, 95%CI = 1.01–1.10, Table S7). However, in either univariate (HR = 1.09, 95%CI = 1.04–1.15, Fig. 5H) or multivariate (HR = 1.08, 95%CI = 1.01–1.17, Table S7) analysis, the significant correlation between the SCS score and OS only existed in the subgroup of patients who underwent ADJC. In terms of the role of the SCS score in predicting chemotherapy benefit, we redefined the cutoff value of the SCS score based on the ROC curve to measure the accuracy of the SCS score in identifying the SCS2 subtype. As expected, only patients with a low SCS score could benefit from ADJC (HR = 0.61, 95%CI = 0.40–0.93, Fig. 5I). In conclusion, the above results revealed that the SCS score is a useful biomarker for predicting the prognosis and chemotherapy efficacy of colon cancer patients, and has good clinical transformation value.

### 3.6 Validation of the SCS score in TCGA‐COAD and SYSUCC cohorts

**Fig. 6:**
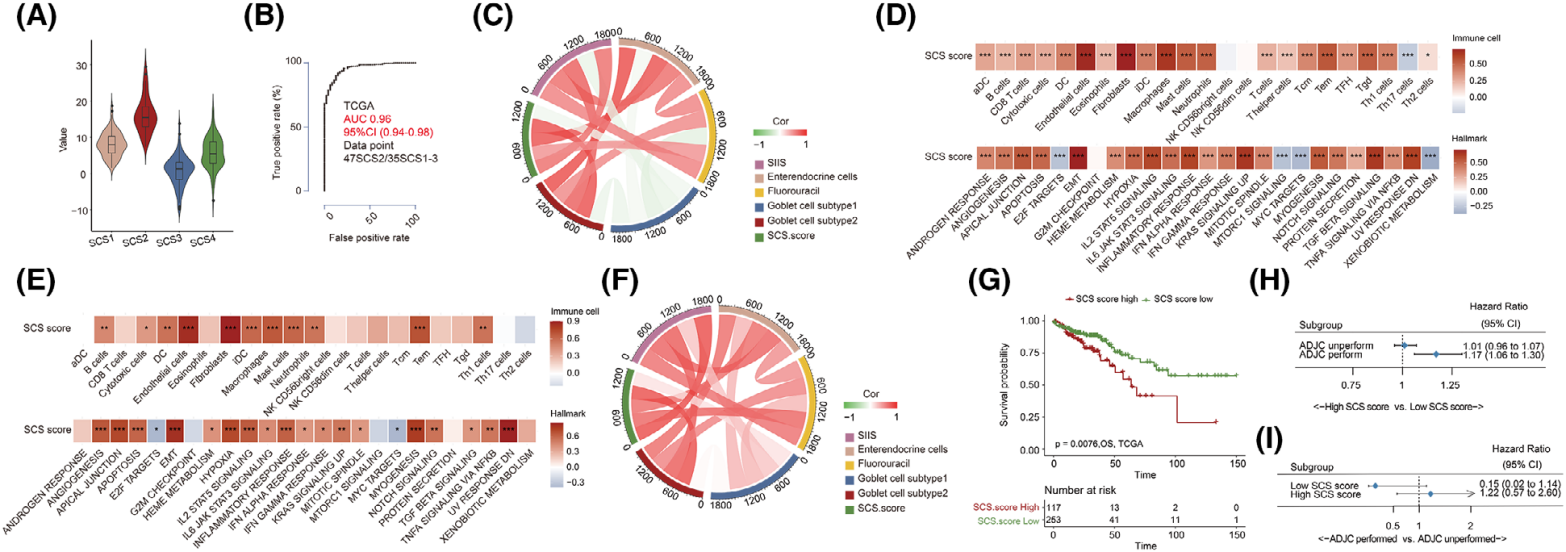
Validation of the SCS score in the TCGA‐COAD and SYSUCC cohort. (A) Violin plot of SCS score values in four SCS subtypes in the TCGA‐COAD cohort (382 patients). Boxes inside the violins represent 25–75% of values, lines in boxes represent median values, whiskers represent 1.5 interquartile ranges, and black dots represent outliers. (B) Receiver operating characteristics curve of the SCS score model in the TCGA‐COAD cohort (382 patients). (C) The correlation chord chart showing the mutual correlation between SIIS value, PCA scores of secretory cells, SCS score, and predicted AUC value of fluorouracil in the TCGA‐COAD cohort (382 patients). (D) Correlation matrix showing the correlations of PCA scores of tumor microenvironment cells (upper) and hallmark pathways (bottom) with the SCS score in the TCGA‐COAD cohort (382 patients). Pearson's correlation test was used to determine significance. (E) The correlation chord chart showing the mutual correlation between SIIS value, PCA scores of secretory cells, SCS score, and predicted AUC value of fluorouracil in the SYSUCC cohort (30 patients). Pearson's correlation test was used to determine significance. (F) Correlation matrix showing the correlations of PCA scores of tumor microenvironment cells (upper) and hallmark pathways (bottom) with the SCS score in the SYSUCC cohort (30 patients). (G) Kaplan–Meier curves of overall survival according to SCS score in the TCGA‐COAD cohort (370 patients); (H) Forest plots of associations between the SCS score and overall survival in subgroups stratified by adjuvant chemotherapy conduction of TCGA‐COAD cohort (323 patients). Unadjusted hazard ratios (boxes) and 95% confidence intervals (horizontal lines) are depicted. (I) Forest plots of benefits of adjuvant chemotherapy in different SCS score groups in the TCGA‐COAD cohort (323 patients). Unadjusted hazard ratios (boxes) and 95% confidence intervals (horizontal lines) are depicted. *P < 0.05, **P < 0.01, ***P < 0.001; ADJC, adjuvant chemotherapy; AUC, area under ROC curve; CI, confidence interval; EMT, epithelial–mesenchymal transition; OS, overall survival; RFS, relapse‐free survival; SCS, secretory cell subtype; SIIS, stromal cell infiltration intensity.

To confirm the clinical value and biological implications of the SCS score, we explored the role of the SCS score in TCGA‐COAD and SYSUCC cohorts. The results showed that the distribution of SCS scores among different SCS subtypes in the TCGA‐COAD cohort was consistent with that in the meta‐GEO cohort (Fig. 6A), and the AUC value of SCS score in identifying patients within the SCS2 subtype was 0.96 in the TCGA‐COAD cohort (Fig. 6B). Moreover, consistent with the results of the meta‐GEO cohort, the positive correlations of SCS score with the activation levels of stromal‐related and inflammation‐related pathways; the PCA scores of enteroendocrine cells, subtype 2 goblet cell, stromal cells, and myeloid cells, and the AUC values of fluorouracil response were all observed in both the TCGA‐COAD (Fig. 6C,D) and SYSUCC (Fig. 6E,F) cohorts. Of note, the elevation of SCS score in MSI‐H patients was also observed in the TCGA‐COAD cohort (Fig. S4B). Further survival analyses of the TCGA‐COAD cohort also showed that a higher SCS score was significantly associated with a higher mortality rate (cutoff determined by survminer package, HR = 2.05, 95%CI = 1.23–3.39, Fig. 6G); however, in the ADJC subgroup, the SCS score was significantly correlated with OS in both univariate (HR = 1.17, 95%CI = 1.06–1.30, Fig. 6H) and multivariate Cox regression (HR = 1.26, 95%CI = 1.09–1.46, Table S8) as a continuous variable. Patients in the high SCS score group were also unable to benefit from ADJC (cutoff determined by ROC curve analysis, HR = 1.22, 95%CI = 0.57–2.60, Fig. 6I). These results suggest strong reproducibility for the SCS score model.

### 3.7 Relationship between the SCS subtype, SCS score, and immunotherapy response

**Fig. 7:**
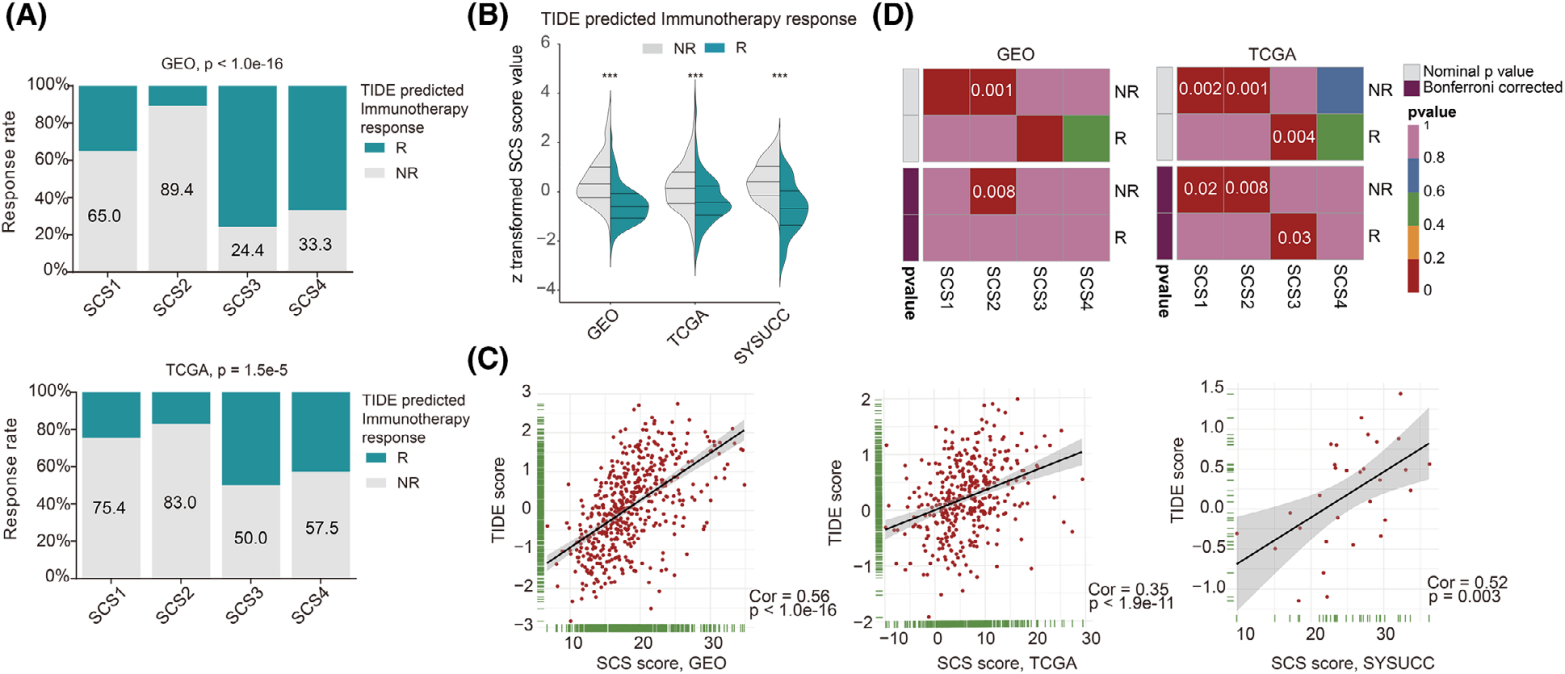
The associations between SCS score and immunotherapy benefits. (A) Bar charts summarizing the proportions of patients with immunotherapy response and those with nonresponse predicted by TIDE algorithm within and across SCS subtypes in the GSE39582 (upper, 502 patients) and TCGA‐COAD cohorts (down, 382 patients). (B) Violin plot of SCS score values in immunotherapy response and nonresponse groups predicted by TIDE algorithm in GSE39582 (502 patients), TCGA‐COAD (382 patients), and SYSUCC (30 patients) cohorts. (C) Scatter plots showing the correlations between SCS score value and TIDE score in the GSE39582 (502 patients), TCGA‐COAD (382 patients), and SYSUCC (30 patients) cohorts. (D) Heatmaps showing the comparison of the similarity between SCS subtypes and immunotherapy response of IMvigor210 dataset in both GSE39582 (502 patients) and TCGA‐COAD cohorts (382 patients) revealed by SubMap analysis. NR, nonresponse; R, response; SCS, secretory cell subtype; TIDE, tumor immune dysfunction and exclusion.

Patients with the TME feature of the ‘immune‐excluded’ phenotype reportedly lack of response to PD‐L1 blockade [23]. Since the TME of the SCS2 subtype was also close to the ‘immune‐excluded’ phenotype, we wondered whether the SCS subtype and SCS score model were associated with the likelihood of responding to immunotherapy. Here, we used the TIDE algorithm to predict the likelihood of a response to immunotherapy. As expected, SCS2 patients may be more unlikely to respond to immunotherapy than other SCS subtypes, with the highest nonresponse rates in both GSE39582 (Fig. 7A, top) and TCGA‐COAD cohorts (Fig. 7A, bottom). Consistently, in the GSE39582, TCGA‐COAD, and SYSUCC cohorts, the SCS score was significantly upregulated in the predicted immunotherapy nonresponsive group (Fig. 7B) and was significantly positively correlated with the TIDE score (Fig. 7C). In addition to the TIDE prediction, we used SubMap to compare the expression profiles of the SCS subtypes we defined with the published dataset containing pretreatment transcriptome data of 217 metastatic urothelial cancer patients who underwent anti‐PD‐L1 treatment. We found that samples of the SCS2 subtype shared significant similarity in marker gene expression with samples of the anti‐PD‐L1 nonresponsive group (Fig. 7D), which further indicates that SCS2 patients may not be candidates who could benefit from ICB (adjusted *P*‐value = 0.008).

### 3.8 The candidate driver genes for fluorouracil resistance in samples of SCS2 subtype

**Fig. 8:**
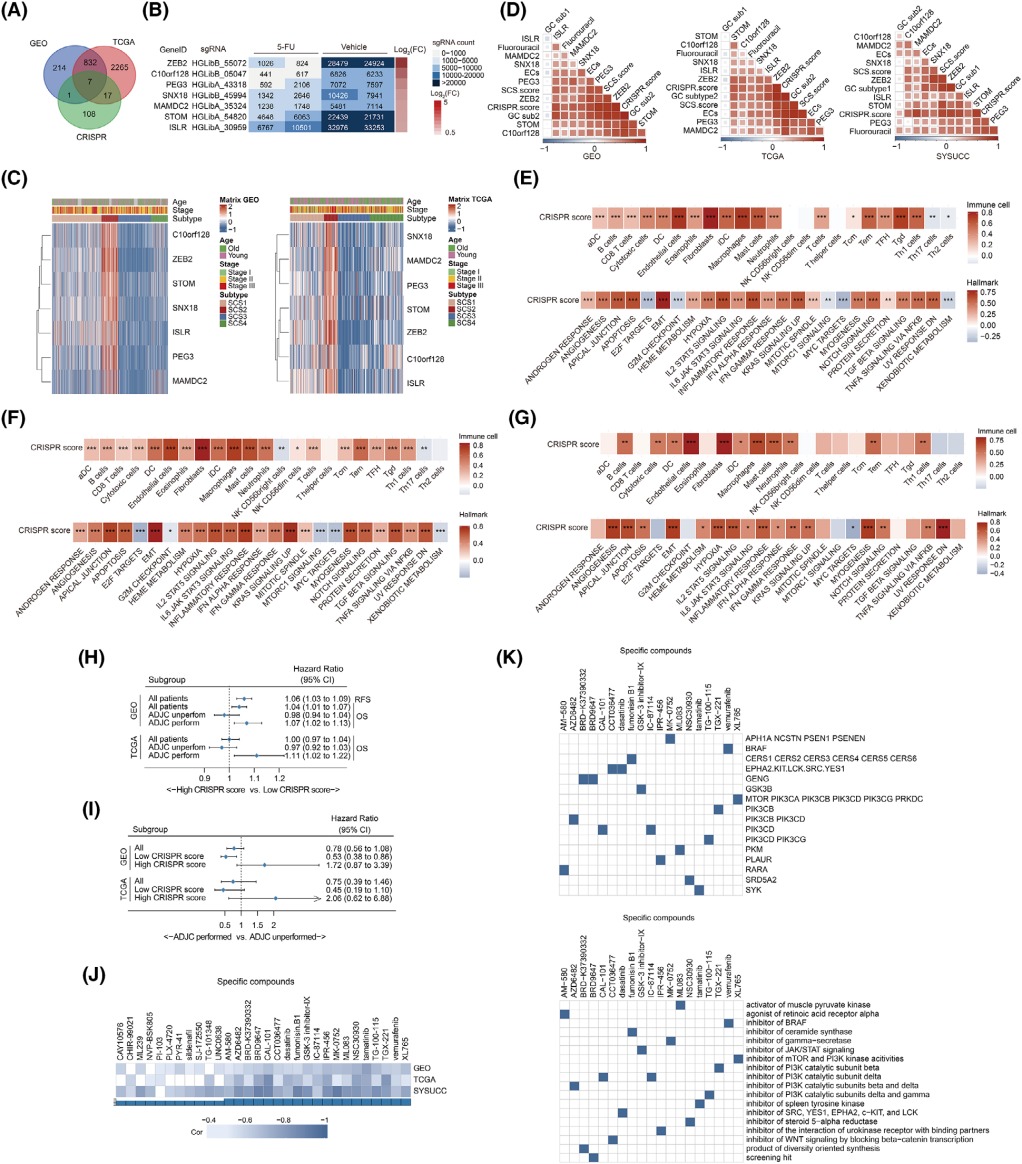
Screening of driver genes and candidate targets or compounds. (A) Venn plot showing deferentially enriched genes in three sets as following: genes upregulated in SCS2 subtype in the GSE39582 cohort (502 patients), genes upregulated in SCS2 subtype in the TCGA‐COAD (382 patients) cohort, candidate driver genes screened out by CRISPR library. (B) Heatmap showing the counts of sgRNA‐representing genes that mediating 5‐FU resistance. (C) Heatmap shows the expression of intersection genes in four SCS subtypes in the meta‐GEO (left, 990 patients) and TCGA‐COAD cohorts (right, 382 patients). Cohort details and SCS subtypes are used as sample annotations of the heatmaps. (D) Heatmaps of the correlation between SCS score, PCA scores of secretory cells, expression of intersection genes, CRISPR score, and predicted AUC value of fluorouracil in the GSE39582 (left, 502 patients), TCGA‐COAD (middle, 382 patients), and SYSUCC (right, 30 patients) cohorts. (E–G) correlation matrix showing the correlations of PCA scores of tumor microenvironment cells (upper) and hallmark pathways (bottom) with the CRISPR score in the GSE39582 (E, 502 patients), TCGA‐COAD (F, 382 patients), and SYSUCC (G, 30 patients) cohorts. Pearson's correlation test was used to determine significance. (H) Forest plots of associations between the CRISPR score and patient survival in various subgroups in the meta‐GEO (RFS analysis, 990 patients and OS analysis, 678 patients) and TCGA‐COAD (370 patients) cohorts. Unadjusted hazard ratios (boxes) and 95% confidence intervals (horizontal lines) are depicted. (I) Forest plots of benefits of adjuvant chemotherapy in different CRISPR score group in the GSE39582 (502 patients) and TCGA‐COAD (323 patients) cohorts. Unadjusted hazard ratios (boxes) and 95% confidence intervals (horizontal lines) are depicted. (J) Heatmaps showing the correlation between SCS score and predicted AUC of compounds based on CTRP database analysis in the GSE39582 (502 patients), TCGA‐COAD (382 patients), and SYSUCC (30 patients) cohorts. (K) Heatmap showing the gene targets(upper) and mechanisms of the action (down) of each compound. *P < 0.05, **P < 0.01, ***P < 0.001; ADJC, adjuvant chemotherapy; CI, confidence interval; EMT, epithelial–mesenchymal transition; FC, fold change; OS, overall survival; RFS, relapse‐free survival; SCS, secretory cell subtype.

In previous studies, we identified several driver genes mediating chemoresistance of colon cancer through CRISPR‐based genome‐wide loss‐of‐function screening in SW480 cells [15]. To explore whether these driver genes were involved in mediating fluorouracil resistance caused by enteroendocrine cells and subtype 2 goblet cell enrichment, we first analyzed the DEGs (Table S9) between SCS2 (highest abundance of enteroendocrine cells and subtype 2 goblet cells with the lowest fluorouracil response rate) and SCS3 (lowest enteroendocrine cells and subtype 2 goblet cells with the highest fluorouracil response rate) in the GSE39582 and TCGA‐COAD cohorts and then obtained the intersection of DEGs and the set of potential driving genes screened by the CRISPR library. As a result, we identified seven core genes whose sgRNA was significantly increased in fluorouracil‐treated survival cell populations and whose expression was significantly increased in the SCS2 subtype in both the meta‐GEO and TCGA‐COAD cohorts (Fig. 8A–C). We defined a new variable named the CRISPR score, the value of which was obtained by summing the expression values of these seven genes. The correlation analysis revealed that (Fig. 8D–G) the CRISPR score was also significantly positively associated with the PCA score of enteroendocrine cells, subtype 2 goblet cells, multiple stromal and myeloid cells, and stromal pathways, estimated AUC of fluorouracil response, and SCS score value in GSE39582, TCGA‐COAD, and SYSUCC cohorts. Clinical analyses further demonstrated that patients with high CRISPR scores exhibited poorer prognoses than those with low CRISPR scores for OS in patients who received ADJC (CRISPR score was analyzed as a continuous variable, Fig. 8H). Similarly, we also found that ADJC performance increased the risk of death in the high CRISPR score subgroup (cutoff determined by the ROC curve, Fig. 8I). Collectively, based on the results from the analysis of these real‐world cohorts, we believe that these seven genes may drive chemoresistance in SCS2 patients in a highly coordinated manner.

### 3.9 Potential compounds for chemosensitization in patients within the SCS2 subtype

As chemotherapy alone might not benefit patients with high enrichment levels of enteroendocrine cells and subtype 2 goblet cells, we explored potential compounds that could be used for chemosensitization in patients with the SCS2 subtype. By analyzing the correlations between the AUC of compounds in the CTRP database and SCS score in the GSE39582, TCGA‐COAD, and SYSUCC cohorts (Table S10), we identified 19 compounds whose correlation coefficients with SCS score were less than −0.5 in three cohorts at the same time (Fig. 8J). Further analysis of gene targets (Fig. 8K, top) and the activity (Fig. 8K, bottom) of these compounds showed that six were inhibitors of members of the PI3K kinase family. Among them, XK765 targets a variety of PI3K catalytic subunits; both TGX‐221 and AZD6482 inhibit the activity of PIK3CB, whereas AZD6482, CAL‐101, and TG‐100‐115 mainly inhibit the activity of PIK3CD. These findings provide a new perspective for developing effective chemosensitizing treatment strategies in SCS2 patients.

## 4 Discussion

The role of secretory cells in the mucosa of colon cancer during tumorigenesis and progression remains unclear, and there are contradictions between existing research conclusions. Meanwhile, although mounting evidence has revealed complex interactions between secretory cells and immune cells involved in the pathogenesis of intestinal inflammatory diseases [5, 6], the relationship between secretory cells and TME heterogeneity has not been investigated. In this study, by using PCA algorithm, we analyzed the expression of gene markers of enteroendocrine cells, subtype 1 goblet cells and subtype 2 goblet cells proposed by Gao et al. [11] in colon cancer tissue, and identified novel molecular subtypes of colon cancer that were significantly correlated with different clinical outcomes, TME characteristics, and biological pathways based on PCA scores of secretory cell signatures using consensus clustering. Among them, the SCS2 subtype, which was highly enriched with enteroendocrine cell and subtype 2 goblet cell signatures, was characterized by the highest PCA score of stromal cell and stromal pathway signatures and was mainly concentrated in the CMS4 type that displayed mesenchymal characteristics [27]. In line with this, patients with SCS2 had the worst RFS, worst OS in patients who were administered chemotherapy, lowest fluorouracil response rate, and might have a low likelihood of responding to ICB treatment based on the TIDE algorithm and SubMap analysis. These observations are consistent with those of our previous studies, which showed that the stromal pathway activation level was associated with negative chemotherapy and immunotherapy outcomes [14, 30, 31]. However, compared with SCS2, the SCS3 subtype (featured by lowest enrichment levels of enteroendocrine cell and subtype 2 goblet cell signatures) and SCS4 subtype (featured by subtype 1 goblet cell signature enrichment) had significantly higher fluorouracil response rates and predicted efficiency of ICB therapy. Meanwhile, SCS4 was mainly concentrated in CMS3 (metabolic disorders) [27], the results of which were also consistent with the positive correlations between PCA scores of subtype 1 goblet cell and metabolic pathways. Previous studies on the role of goblet cells in driving tumor progression have drawn contradictory conclusions. For example, although overexpression of MUC2, the marker of goblet cells, was shown to be associated with increased colon cancer risk and tumor development [33], some studies in the literature have also indicated that MUC2 secreted by goblet cells can exert an anti‐cancer effect by protecting the intestinal mucosa [33] and the loss of MUC2 expression was a predictor of adverse outcomes [34]. Based on the results of this study, we speculated that the reason for the inconsistent research findings may be attributed to the heterogeneity between subtype 1 and subtype 2 goblet cells. Although both the SCS2 subtype and SCS4 subtype showed high enrichment of goblet cell markers such as TFF3 and MUC2 after adjusting of tumor purity data (Fig. S5), only the PCA score of subtype 2 goblet cell signature and the SCS2 subtype were linked with worse clinical behavior and enrichment levels of stromal cell‐ and stromal pathway‐related signatures. Hence, these results also suggested that traditional detection of goblet cells in colon cancer tissue by morphology‐based H&E staining or immunohistochemical or immunofluorescence staining of MUC2 and other molecular markers [7, 35] has great limitations. Finally, in the multi‐omic analysis of different SCS subtypes, we were surprised to find that although the activation degree of KRAS pathway at the transcriptome level was significantly increased in SCS2 subtype compared with other subtypes, the somatic mutation of KRAS was significantly enriched in SCS4 subtype and the PCA score of subtype 1 goblet cell signature in the KRAS mutant group was also significantly higher compared with that in the KRAS wild‐type group. We hypothesized that this result may be due to the presence of other factors that inhibit KRAS signaling activation in the SCS4 subtype [36]. Therefore, the specific link between KRAS mutation and goblet cell accumulation and differentiation in colon cancer needs to be further examined in future studies.

Since our study revealed that the SCS subtype is one of the sources of tumor heterogeneity, it is of great clinical significance to establish an easy‐to‐use scoring tool to identify patients with the SCS2 subtype indicating with poor prognosis and chemotherapy resistance. Therefore, we identified seven candidate genes through a series of dimensionality reduction steps and established a transcriptome‐based model, namely ‘SCS score’. The SCS score can accurately define the SCS2 subtype. Subsequent clinical analysis showed that the SCS score is not only an independent prognostic factor but can also discriminate patients who can benefit from ADJC. Positive correlations between SCS score and PCA scores of enteroendocrine cells, subtype 2 goblet cells, stromal and myeloid cells, and stromal pathways, and predicted AUC values of fluorouracil response were also confirmed in the meta‐GEO, TCGA‐COAD, and SYSUCC cohorts. In conclusion, these findings not only revealed that the SCS score has excellent reproducibility and clinical applicability but also further validated our hypothesis that the secretory cell enrichment pattern could be applied in clinical practice to define TME characteristics and guide treatment decisions.

To date, the molecular mechanisms by which secretory cells cause chemotherapy resistance have rarely been studied. Previous studies have shown that the mucosa, which surrounds the tumor cells, might function as a physical barrier to the delivery of chemotherapy [3] and that compressing forces (solid stress) exerted by the bulky mucinous tumor on the penetrating vascular system might reduce the extent of drug delivery to the tumor [37]. However, in the current study, we identified seven potential candidate genes that were highly expressed in the SCS2 subtype, which might serve as the core genes driving chemoresistance related to enteroendocrine cell and subtype 2 goblet cell enrichment in a highly coordinated manner based on the results of genome‐wide CRISPR screening. Among these genes, ZEB2 was reported to mediate crosstalk between TGFβ signaling and the EMT pathway [38], which further emphasizes the tumor‐promoting activity of TGFβ signaling and the therapeutic role of TGFβ blockade in the SCS2 subtype. However, we also screened for potential compounds that could be used for chemosensitization in SCS2 patients based on the CTRP database, and several drugs targeting PI3K catalytic subunits were identified. Coincidentally, previous studies have also demonstrated that the PI3K/AKT pathway is a key downstream pathway involved in TFF3‐related carcinogenic processes [39]. Therefore, we propose that PIK3 inhibitors may be effective candidates for the treatment of patients with SCS2. Accordingly, systematic preclinical studies investigating the efficiency of PIK3 inhibitors as combinatorial targeted therapies are warranted.

Our study has some limitations. First, the enrichment level of secretory cells in the tumor tissue was only represented by a simple score calculated based on bulk transcriptome data. A visual verification was not performed. Second, retrospective studies inevitably have selection bias and the cutoff values of continuous variables need to be standardized in future prospective studies. Finally, due to the CRISPR screen data used in this study were not being derived from secretory cell lines, the specific function of the seven chemoresistance driver genes in SCS2 tumor tissues and whether these genes can mediate chemoresistance in colon cancer cells with high expression of enteroendocrine‐ and subtype2 goblet cell‐related molecular features still need more in‐depth exploration.

## 5 Conclusions

This study comprehensively uncovered the heterogeneity of the clinical behavior, TME, and biological behavior related to the enrichment of secretory cell marker transcriptional expression. Moreover, The SCS score is an easily accessible model that can be used for identification of SCS2 patients, a group of patients with special clinical features. We suggest that the SCS score can be used as an important supplement to routine histopathological assessment by clinicians to guide decision‐making in regulating multimodal treatment of patients with colon cancer in the near future.

## Conflict of interest

The authors declare no conflict of interest.

## Author contributions

Study design and supervision: WL; Public data collection: RZ and LL; Clinical sample collection: XS; Data analysis and visualization: RZ, LL, and SX; Independent result validation: YZ, ZL, DZ, HS, and, JW; Result interpretation: LW, MS, JB, and YL; Manuscript draft: RZ, LL, and SX; Manuscript review and revise: WL.

## Supporting information


**Fig. S1.** Workflow diagram of this study.

**Fig. S2.** Violin plot of SIIS values in four SCS subtypes in the meta‐GEO and TCGA‐COAD cohorts. Violin plot of SIIS values in four SCS subtypes in the meta‐GEO (left, 990 patients) and TCGA‐COAD (right, 382 patients) cohorts. Boxes inside the violins represent 25–75% of values, lines in boxes represent median values, whiskers represent 1.5 interquartile ranges, and black dots represent outliers.

**Fig. S3.** SCS subtype‐related genomic alterations in MSI‐H and MSS/MSI‐L cohorts. (A‐B) Oncoprints depicted the somatic mutations of significant mutated genes in the context of four SCS subtypes in MSI‐H (A, 65 patients) and MSS/MSI‐L (B, 274 patients) patients of the TCGA‐COAD cohort. Cohort details and SCS subtypes are used as sample annotations of the heatmaps. (C‐D) Box plots show the distribution of PCA scores of secretory cells and predicted AUC value of fluorouracil in different mutation status of gene combination in MSI‐H (C, 65 patients) and MSS/MSI‐L patients (D, 274 patients). Boxes represent 25–75% of values, lines in boxes represent median values, whiskers represent 1.5 interquartile ranges, and black dots represent outliers. ***p < 0.001; SCS, secretory cell subtype.

**Fig. S4.** Violin plot of SCS score value in patients with different MMR and MSI status. Violin plot of SCS score value in patients with different MMR (left, 458 patients) and MSI status (right, 371 patients). Boxes inside the violins represent 25–75% of values, lines in boxes represent median values, whiskers represent 1.5 interquartile ranges, and black dots represent outliers.

**Fig. S5.** Box plot of the distribution of gene expression of TFF3 and MUC2 adjusted by tumor purity among four SCS subtypes in the TCGA‐COAD cohort. Box plot of the distribution of gene expression of TFF3 and MUC2 adjusted by tumor purity among four SCS subtypes in the TCGA‐COAD cohort (304 patients). Boxes inside the violins represent 25–75% of values, lines in boxes represent median values, whiskers represent 1.5 interquartile ranges, and black dots represent outliers. *SCS, secretory cell subtype*.

**Table S1.** Basic information of gene expression profiling series.

**Table S2.** Patients' basic characteristics.

**Table S3.** The correlation between the abundance of secretory cells and KEGG items.

**Table S4.** Significantly varied methylation genes.

**Table S5.** Differentially expressed genes in SCS2 subtype.

**Table S6.** The summaries for genes included in the SCS score model.

**Table S7.** Multivariate survival analyses of SCS score and clinical variables in the meta‐GEO cohort.

**Table S8.** Multivariate survival analyses of SCS score and clinical variables in the TCGA‐COAD cohort.

**Table S9.** Differentially expressed genes between SCS2 and SCS3.

**Table S10.** The correlation between SCS score and AUC of compounds in CTRP database in GSE39582.

## Data Availability

The public data used in this study are available at: GSE17536 (http://www.ncbi.nlm.nih.gov/geo/query/acc.cgi?acc=GSE17536); GSE33113 (http://www.ncbi.nlm.nih.gov/geo/query/acc.cgi?acc=GSE33113); GSE37892 (http://www.ncbi.nlm.nih.gov/geo/query/acc.cgi?acc=GSE37892); GSE38832 (http://www.ncbi.nlm.nih.gov/geo/query/acc.cgi?acc=GSE38832); GSE39582 (http://www.ncbi.nlm.nih.gov/geo/query/acc.cgi?acc=GSE39582); TCGA‐COAD (https://xenabrowser.net/datapages/?cohort=TCGA); Colon Cancer (COAD); imvigor210 r package ‘IMvigor210CoreBiologies’. The SUSYCC colon cancer dataset generated and analyzed during the current study are not publicly available but are available from the corresponding author on reasonable request.
