# Supplementary material for: Expression pattern of secretory‐cell‐related transcriptional signatures in colon adenocarcinomas defines tumor microenvironment characteristics and correlates with clinical outcomes
Source: Mol Oncol. 2022 Nov 22;17(3):499–517. doi: 10.1002/1878-0261.13338 (PMC9980301; doi:10.1002/1878-0261.13338)
Supplement: Supplementary file 1 — Fig. S1. Workflow diagram of this study. [file MOL2-17-499-s003.pdf]

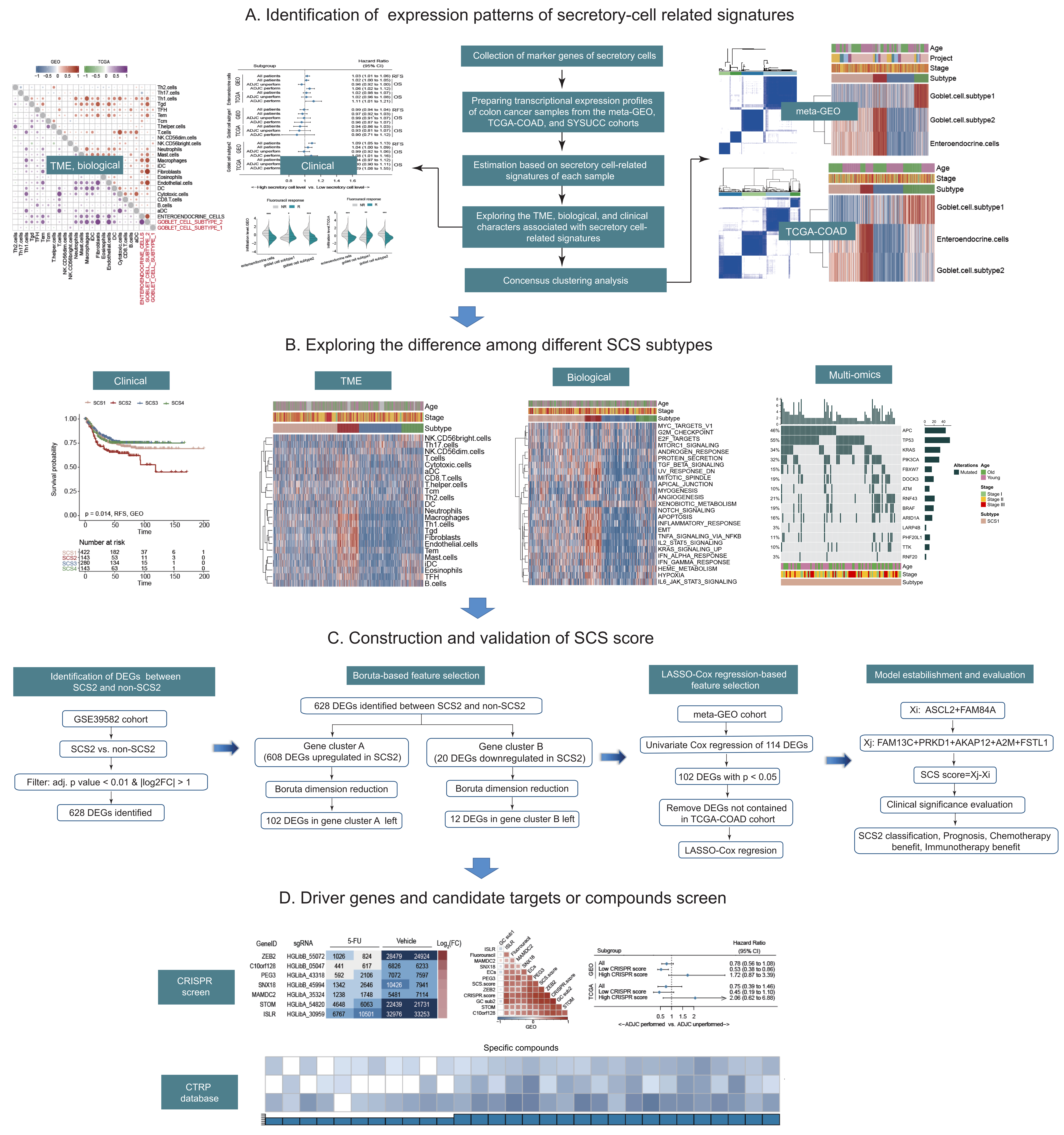
A. Identification of expression patterns of secretory-cell related signatures
Collection of marker genes of secretory cells
Preparing transcriptional expression profiles of colon cancer samples from the meta-GEO, TCGA-COAD, and SYSUCC cohorts
Estimation based on secretory cell-related signatures of each sample
Exploring the TME, biological, and clinical characters associated with secretory cell-related signatures
Concensus clustering analysis
TME, biological
Clinical
meta-GEO
TCGA-COAD
Age
Project
Stage
Subtype
Goblet.cell.subtype1
Goblet.cell.subtype2
Enteroendocrine.cells
B. Exploring the difference among different SCS subtypes
Clinical
TME
Biological
Multi-omics
SCS1
SCS2
SCS3
SCS4
Survival probability
p = 0.014, RFS, GEO
Time
Number at risk
C. Construction and validation of SCS score
Identification of DEGs between SCS2 and non-SCS2
GSE39582 cohort
SCS2 vs. non-SCS2
Filter: adj. p value < 0.01 & |log2FC| > 1
628 DEGs identified
Boruta-based feature selection
628 DEGs identified between SCS2 and non-SCS2
Gene cluster A (608 DEGs upregulated in SCS2)
Gene cluster B (20 DEGs downregulated in SCS2)
Boruta dimension reduction
Boruta dimension reduction
102 DEGs in gene cluster A left
12 DEGs in gene cluster B left
LASSO-Cox regression-based feature selection
meta-GEO cohort
Univariate Cox regression of 114 DEGs
102 DEGs with p < 0.05
Remove DEGs not contained in TCGA-COAD cohort
LASSO-Cox regresion
Model estabilishment and evaluation
Xi: ASCL2+FAM84A
Xj: FAM13C+PRKD1+AKAP12+A2M+FSTL1
SCS score=Xj-Xi
Clinical significance evaluation
SCS2 classification, Prognosis, Chemotherapy benefit, Immunotherapy benefit
D. Driver genes and candidate targets or compounds screen
CRISPR screen
GeneID
sgRNA
5-FU
Vehicle
Log2(FC)
ZEB2 HGLibB_55072 1026 824 28479 24924
C10orf128 HGLibB_05047 441 617 6826 6233
PEG3 HGLibA_43318 592 2106 7072 7597
SNX18 HGLibB_45994 1342 2646 10426 7941
MAMDC2 HGLibA_35324 1238 1748 5481 7114
STOM HGLibA_54820 4648 6063 22439 21731
ISLR HGLibA_30959 6767 10501 32976 33253
Subgroup
Hazard Ratio (95% CI)
GEO
All 0.78 (0.56 to 1.08)
Low CRISPR score 0.53 (0.38 to 0.86)
High CRISPR score 1.72 (0.87 to 3.39)
TCGA
All 0.75 (0.39 to 1.46)
Low CRISPR score 0.45 (0.19 to 1.10)
High CRISPR score 2.06 (0.62 to 6.88)
<--ADJC performed vs. ADJC unperformed-->
Specific compounds
CTRP database
