# Supplementary figures and images for: Expression pattern of secretory‐cell‐related transcriptional signatures in colon adenocarcinomas defines tumor microenvironment characteristics and correlates with clinical outcomes
Source: Mol Oncol. 2022 Nov 22;17(3):499–517. doi: 10.1002/1878-0261.13338 (PMC9980301; doi:10.1002/1878-0261.13338)

A

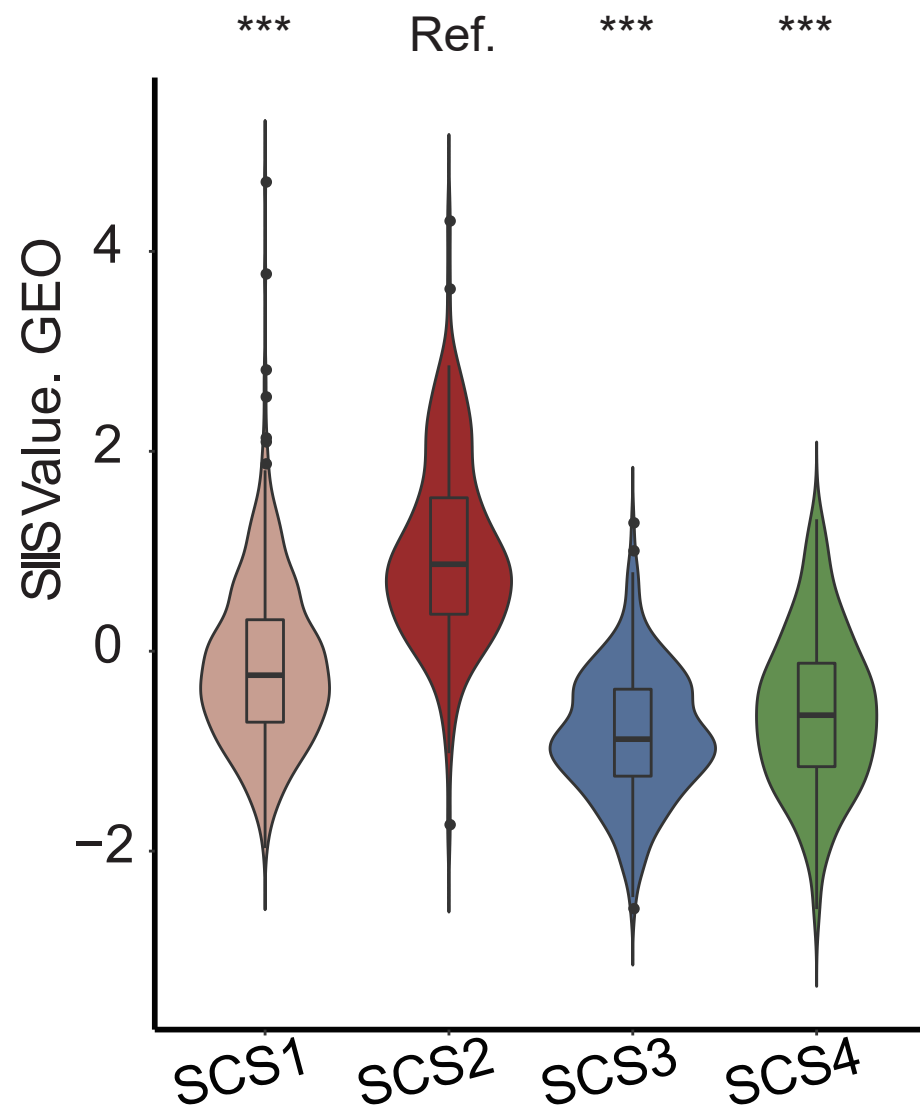

B

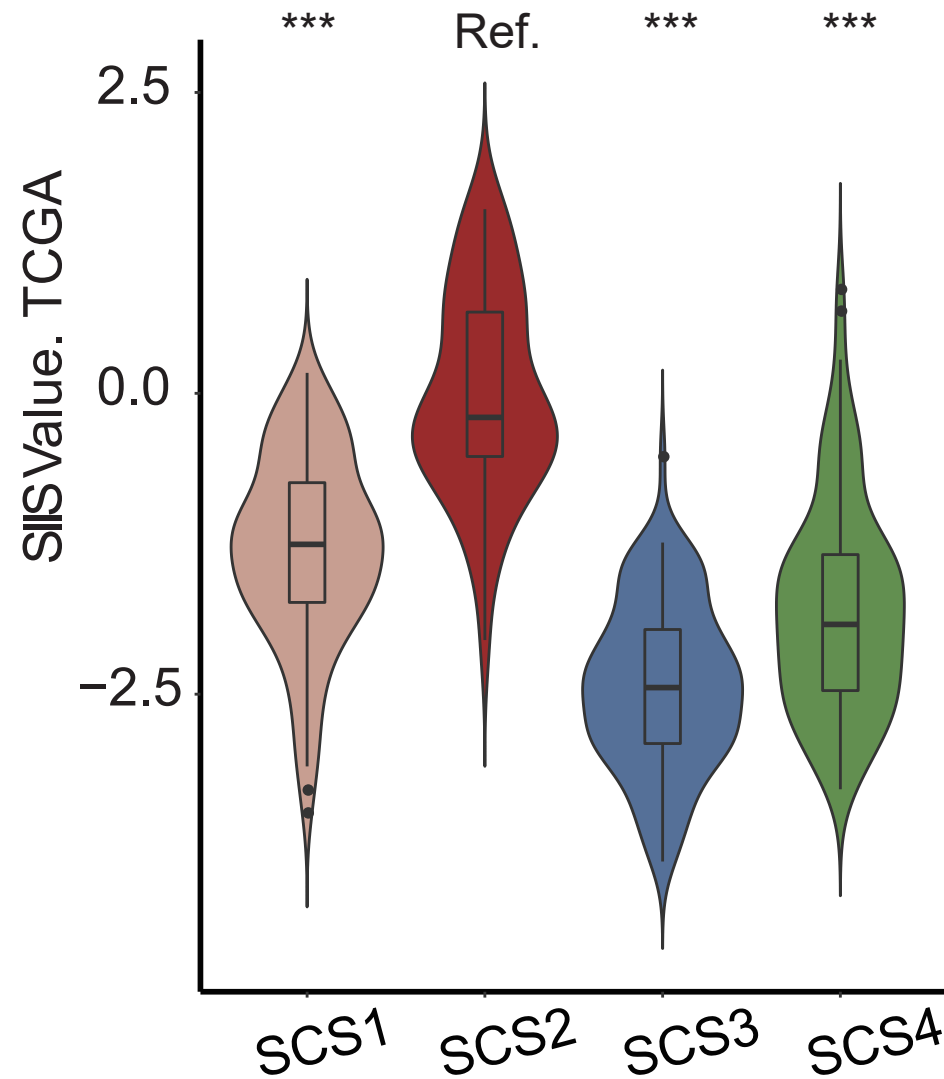

Supplement: Supplementary file 2 — Fig. S2. Violin plot of SIIS values in four SCS subtypes in the meta‐GEO and TCGA‐COAD cohorts. Violin plot of SIIS values in four SCS subtypes in the meta‐GEO (left, 990 patients) and TCGA‐COAD (right, 382 patients) cohorts. Boxes inside the violins represent 25–75% of values, lines in boxes represent median values, whiskers represent 1.5 interquartile ranges, and black dots represent outliers. [file MOL2-17-499-s001.pdf]

A

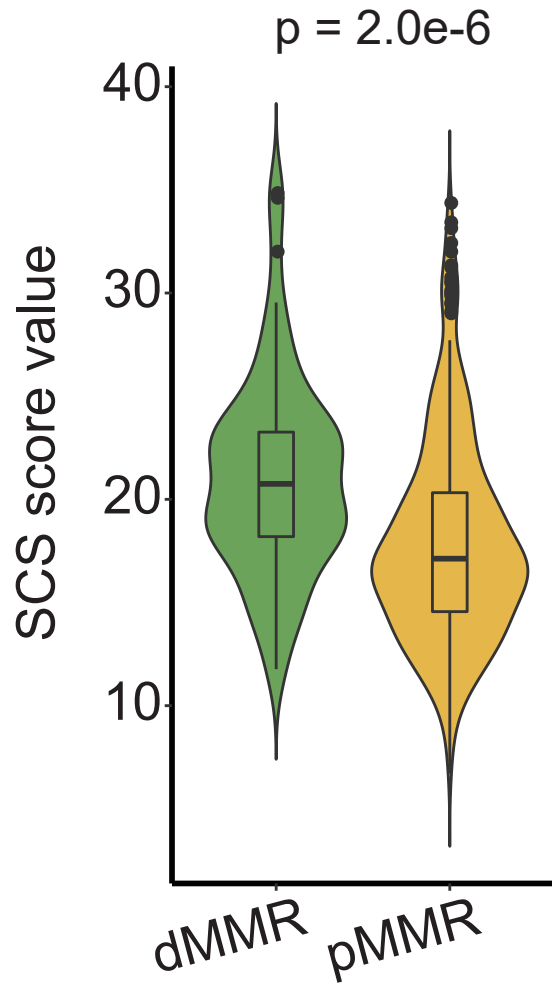

B

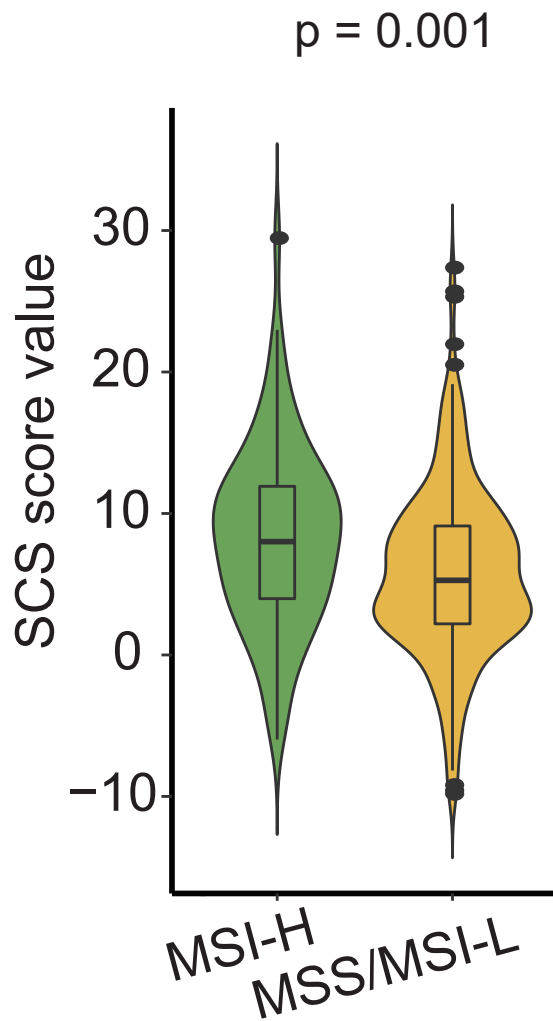

Supplement: Supplementary file 4 — Fig. S4. Violin plot of SCS score value in patients with different MMR and MSI status. Violin plot of SCS score value in patients with different MMR (left, 458 patients) and MSI status (right, 371 patients). Boxes inside the violins represent 25–75% of values, lines in boxes represent median values, whiskers represent 1.5 interquartile ranges, and black dots represent outliers. [file MOL2-17-499-s011.pdf]

Tumor purity adjusted gene expression

SCS1 SCS2 SCS3 SCS4

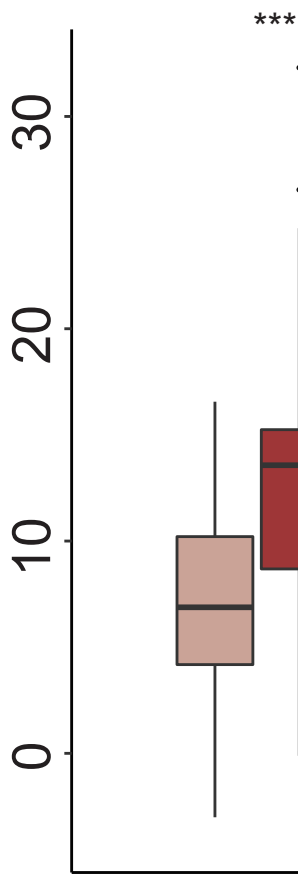

TFF3

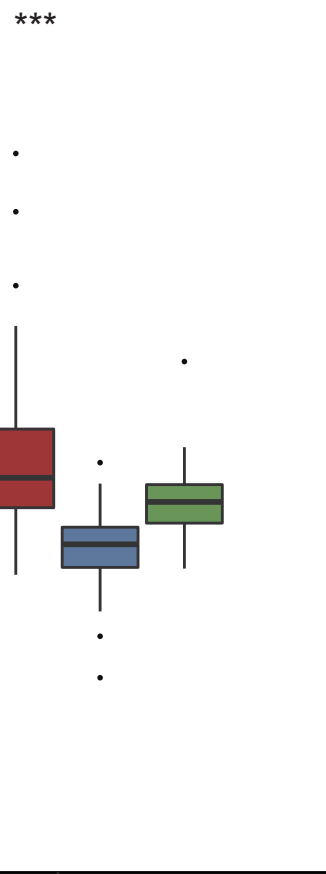

MUC2

Supplement: Supplementary file 5 — Fig. S5. Box plot of the distribution of gene expression of TFF3 and MUC2 adjusted by tumor purity among four SCS subtypes in the TCGA‐COAD cohort. Box plot of the distribution of gene expression of TFF3 and MUC2 adjusted by tumor purity among four SCS subtypes in the TCGA‐COAD cohort (304 patients). Boxes inside the violins represent 25–75% of values, lines in boxes represent median values, whiskers represent 1.5 interquartile ranges, and black dots represent outliers. SCS, secretory cell subtype. [file MOL2-17-499-s010.pdf]
