# Supplementary material for: Expression pattern of secretory‐cell‐related transcriptional signatures in colon adenocarcinomas defines tumor microenvironment characteristics and correlates with clinical outcomes
Source: Mol Oncol. 2022 Nov 22;17(3):499–517. doi: 10.1002/1878-0261.13338 (PMC9980301; doi:10.1002/1878-0261.13338)
Supplement: Supplementary file 3 — Fig. S3. SCS subtype‐related genomic alterations in MSI‐H and MSS/MSI‐L cohorts. (A‐B) Oncoprints depicted the somatic mutations of significant mutated genes in the context of four SCS subtypes in MSI‐H (A, 65 patients) and MSS/MSI‐L (B, 274 patients) patients of the TCGA‐COAD cohort. Cohort details and SCS subtypes are used as sample annotations of the heatmaps. (C‐D) Box plots show the distribution of PCA scores of secretory cells and predicted AUC value of fluorouracil in different mutation status of gene combination in MSI‐H (C, 65 patients) and MSS/MSI‐L patients (D, 274 patients). Boxes represent 25–75% of values, lines in boxes represent median values, whiskers represent 1.5 interquartile ranges, and black dots represent outliers. ***p < 0.001; SCS, secretory cell subtype. [file MOL2-17-499-s009.pdf]

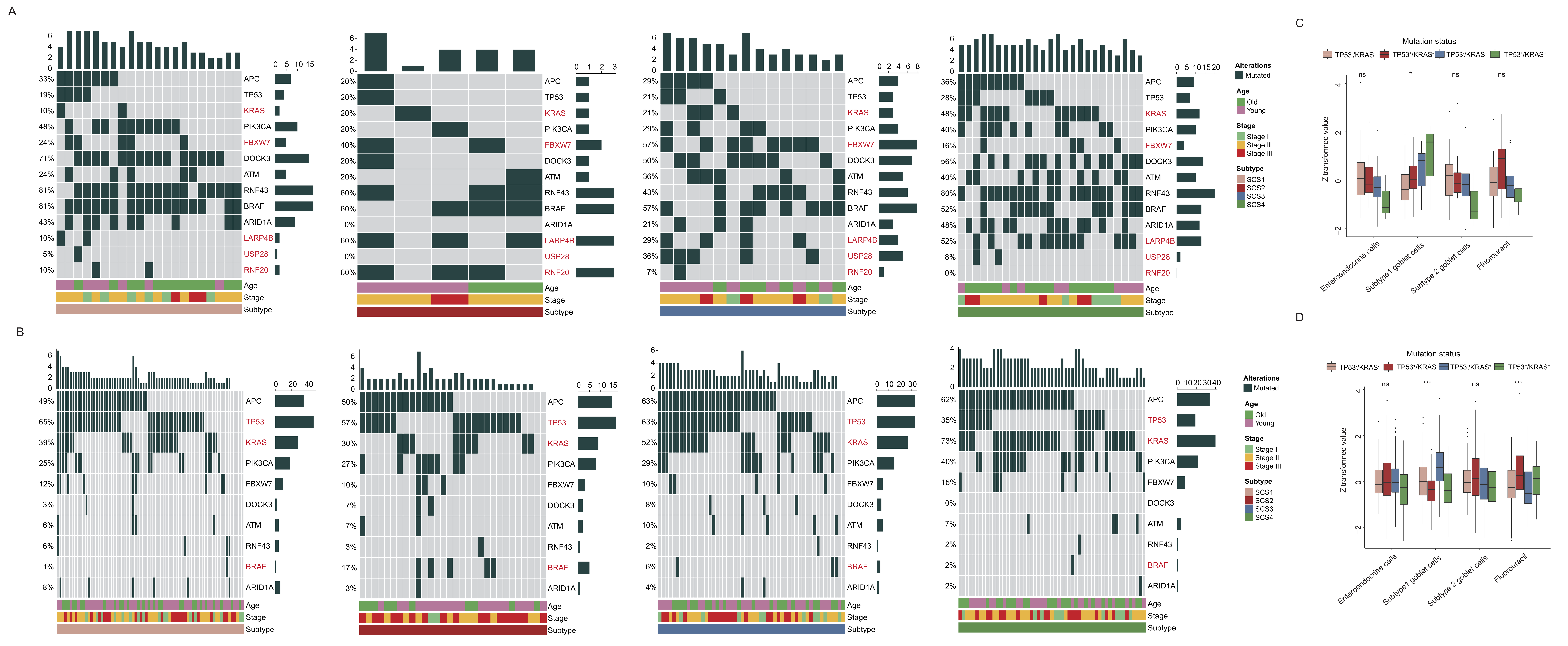

A
B
C
D
APC
TP53
KRAS
PIK3CA
FBXW7
DOCK3
ATM
RNF43
BRAF
ARID1A
LARP4B
USP28
RNF20
Age
Stage
Subtype
Alterations
Mutated
Old
Young
Stage I
Stage II
Stage III
SCS1
SCS2
SCS3
SCS4
Mutation status
Z transformed value
Enteroendocrine cells
Subtype1 goblet cells
Subtype 2 goblet cells
Fluorouracil
ns
